# Microbial diversity and metabolic function in duodenum, jejunum and ileum of emu (*Dromaius novaehollandiae*)

**DOI:** 10.1038/s41598-023-31684-8

**Published:** 2023-03-18

**Authors:** Ji Eun Kim, Hein M. Tun, Darin C. Bennett, Frederick C. Leung, Kimberly M. Cheng

**Affiliations:** 1 Avian Research Centre, Faculty of Land and Food Systems, University of British Columbia, 2357 Main Mall, Vancouver, BC V6T 1Z4 Canada; 2 School of Public Health, Li Ka Shing, Faculty of Medicine, HKU-Pasteur Research Pole, University of Hong Kong, Pok Fu Lam, Hong Kong SAR, China; 3 JC School of Public Health and Primary Care, Faculty of Medicine, Chinese University of Hong Kong, Sha Tin, Hong Kong SAR, China; 4 Animal Science Department, California Polytechnic State University, San Luis Obispo, CA 93407 USA; 5 School of Biological Sciences, Faculty of Science, University of Hong Kong, Pok Fu Lam, Hong Kong SAR, China

**Keywords:** Microbiology, Zoology, Gastroenterology

## Abstract

Emus (*Dromaius novaehollandiae*), a large flightless omnivorous ratite, are farmed for their fat and meat. Emu fat can be rendered into oil for therapeutic and cosmetic use. They are capable of gaining a significant portion of its daily energy requirement from the digestion of plant fibre. Despite of its large body size and low metabolic rate, emus have a relatively simple gastroinstetinal (GI) tract with a short mean digesta retention time. However, little is known about the GI microbial diversity of emus. The objective of this study was to characterize the intraluminal intestinal bacterial community in the different segments of small intestine (duodenum, jejunum, and ileum) using pyrotag sequencing and compare that with the ceca. Gut content samples were collected from each of four adult emus (2 males, 2 females; 5–6 years old) that were free ranged but supplemented with a barley-alfalfa-canola based diet. We amplified the V3-V5 region of 16S rRNA gene to identify the bacterial community using Roche 454 Junior system. After quality trimming, a total of 165,585 sequence reads were obtained from different segments of the small intestine (SI). A total of 701 operational taxonomic units (OTUs) were identified in the different segments of small intestine. Firmicutes (14–99%) and Proteobacteria (0.5–76%) were the most predominant bacterial phyla in the small intestine. Based on species richness estimation (Chao1 index), the average number of estimated OTUs in the small intestinal compartments were 148 in Duodenum, 167 in Jejunum, and 85 in Ileum, respectively. Low number of core OTUs identified in each compartment of small intestine across individual birds (Duodenum: 13 OTUs, Jejunum: 2 OTUs, Ileum: 14 OTUs) indicated unique bacterial community in each bird. Moreover, only 2 OTUs (*Escherichia* and *Sinobacteraceae*) were identified as core bacteria along the whole small intestine. PICRUSt analysis has indicated that the detoxification of plant material and environmental chemicals seem to be performed by SI microbiota, especially those in the jejunum. The emu cecal microbiome has more genes than SI segments involving in protective or immune response to enteric pathogens. Microbial digestion and fermentation is mostly in the jejunum and ceca. This is the first study to characterize the microbiota of different compartments of the emu intestines via gut samples and not fecal samples. Results from this study allow us to further investigate the influence of the seasonal and physiological changes of intestinal microbiota on the nutrition of emus and indirectly influence the fatty acid composition of emu fat.

## Introduction

The gastrointestinal (GI) microbiota has been recognized as an essential component of the intestinal ecosystem, which contributes to the wellbeing, energy metabolism and disease resistance in animals^1–3^.They play a critical role in the health of animals through nutrient utilization, immunological development and other physiological systems^4^. Animals maintain complex and intimate associations with a diverse community of GI microbes^5^. In order to characterize the GI microbiota diversity, earlier studies applied selective and cultivation-based techniques to identify potential pathogenic microbes^6,7^. However, these studies revealed limited number of bacteria communities. Subsequently, the approach of the pyrotag sequencing of 16S rRNA genes and metagenomics has made it possible to better characterize the GI microbiota communities and examine their interaction with host and diet.

Avian represents interesting study models in which to investigate the roles of intestinal microbes in the nutrition, immune function, and development because they have unique diets, physiological traits, and developmental strategies^8^. Moreover, avian has a shorter GI tract and faster digesta transit time less than 3.5 h^9^. This anatomic feature selects a very different intestinal microbiome in avian than mammals^10^. The GI microbiota community of both domestic and wild bird species including chicken, turkey, duck and ratites has been studied by pyrosequencing^2,11–13^.

Most studies of avian species have focused on characterizing the microbiota in the ceca due to its large bacterial diversity^13–16^. Examination of omnivorous avian species shows that *Bacteroidetes* is the dominate phylum in the ceca^15,17–21^. In contrast, *Firmicutes* dominate in the ceca of the ostrich (*Struthio camelus*), Japanese quail (*Cotuenix japonica*), and capercaille (*Tetrao urogallus*), which are predominantly herbivores^14,22^. Research foci have subsequently included the variation in microbial community along the GI tract^23^. Several studies in chickens (*Gallus gallus domestica*) showed that *Lactobacilli* to be the major bacterial population in three segments (duodenum, jejunum, and ileum) of the small intestine, whereas *Clostridium spp*. and *Bacteroides spp*. are dominant in the ceca^17,24^.

Unfortunately, until recently, the lack of standardized protocols in avian microbiota studies and the mainly use of fecal or cloacal samples [25. 26] prevents meaningful comparisons of microbiome across different intestinal segments^23,27^. Moreover, analysis of the small intestine microbiota precludes non-lethal sampling because of its location in the GI tract. As a result, microbiota variation along different segments of the GI tract has only been studied in very few species: chicken^28,29^, turkey (*Meleagris gallopavo*)^30^, hoatzin (*Opisthocomus hoazin*)^31^, kakapo (*Strigops habroptilus*)^32^, and Japanese quail^33,34^.

Emu (*Dromaius novaehollandiae*) is a flightless omnivorous bird native to Australia. Oil extracted from the fat tissue of emu has been traditionally used by aborigines for wound healing. Presently, emu oil is commonly used in cosmetics preparations^35^. Veterinary, alternative and traditional medicine has also included the use emu oil for the treatment of wounds and inflammatory skin conditions.

Despite emu’s behavior of seasonal dietary intake and fat deposition, there is little known about the host interaction with GI microbiota ecosystem. Our previous study has shown that the predominant bacterial phyla is *Bacteroidetes* in emu ceca^13^. The objective of this study is to characterize the bacteria community and predict microbial metabolic function in the three segments (duodenum, jejunum and ileum) of the small intestine (SI) in comparison with that in the ceca^13^ using pyrotag sequencing with 16S rRNA gene.

## Methods

The study was conducted in accordance with the relevant guidelines and regulations. Methods are reported in accordance with ARRIVE guidelines (https://arriveguidelines.org).

### Experimental animals

Along with the emu ceca in our previous study^13^, SI segments (duodenum, jejunum and ileum) were collected from the same four adult emus (2 males, 2 females) at TryHarder Farm (Saskatchewan, Canada) for use in this study. The emus were free-ranged (natural forage) but supplemented with a barley-alfalfa-canola based diet. Detailed rearing and processing procedure can be found in Bennett et al.^13^. The ceca and SI segment samples were collected in early November, just prior to the onset of their breeding season. From past studies^13,36^ seasonal decline in their feed intake should have begun. However, we did not measure individual feed intake. The Ceca and SI samples were frozen immediately after collection and kept at − 80 ℃ until use. The study was approved by the Animal Care and Use Committee at University of British Columbia (Certificate # A10-0106).

### DNA extraction and 16S rRNA gene amplicons

Together with the cecal samples, the SI samples were thawed on ice and the contents were gently scraped from the intestinal wall of each sample. Using the same protocol described by Bennett et al.^13^, genomic DNA was extracted from each of the four duodenal, jejunal and ileal samples using the PowerMax Soil DNA Isolation Kit (Mo Bio Laboratories Inc., Carlsbad, CA) according to the instructions of the manufacturer with 200 mg/sample as starting material. Extracted DNA was amplified by PCR using FastStart high fidelity PCR system (Roche Molecular Diagnostics, Branchburg, NJ, USA). A universal primer set of 341F (5’- ACTCCTACGG GAGGCAGCAG-3’) and 926R (5’- CCGTCAATTCMTTTGAGTTT-3’) was adopted for amplifying the variable region 3 to 5 (V3-V5) of the bacterial 16S rRNA gene. The forward primer bore a multiplex identifier (MID) sequences for sample identification, and the primer set was modified by adding adaptor A and B sequence respectively for pyrotag sequencing. The amplification program consisted of an initial denaturation step at 94 °C for 2 min; 32 cycles of denaturation at 94 °C for 30 s, annealing at 60 °C for 30 s, and elongation at 72 °C for 30 s; and a final extension step at 72 °C for 7 min. The size of the PCR products was confirmed by gel electrophoresis, and then, the PCR products was purified using Invitrogen Purelink Quick Gel Extraction Kit (Invitrogen, Oregon, USA) and were quantified using the Nanodrop (ND-2000) spectrophotometer (Nanodrop Technologies, Wilmington, DE, USA). The sequencing of the 16S rRNA genes was performed by 454 GS Junior (454 Life Sciences—Roche, Branford, CT, USA) according to the manufacturer’s instructions. Tag-encoded pyrosequence data were deposited into NCBI Sequence Read Archive under accession number SRA071216.

### Sequence analysis

Sequencing reads obtained from pyrosequencing were subjected to processing with QIIME (quantitative insights into microbial ecology) 1.8.0 software package^37^ for downstream analysis. For quality trimming, reads are removed as per the following criteria: a mean quality score less than 25, length of < 150 or > 900 bp, without primer sequence, containing ambiguous characters, homopolymer run exceeding 8 nt, or uncorrectable. Based on the barcode sequences, the remaining sequences were de-multiplexed, followed by denoising using DENOISER v. 0.9.1^38^ and removal of chimeric sequences using ChimeraSlayer (http://microbiomeutil.sourceforge.net/). UCLUST (https://drive5.com/usearch/manual/uclust_algo.html) was used to cluster the remaining sequence into Operational Taxonomic Units (OTUs) at 97% sequence similarity. Next, the taxonomy was assigned to each representative sequence of each OTU using Ribosomal Database Project (RDP) classifier 2.0.1^39^. Alignment of the OTU representative sequences was performed using PyNAST with a minimum alignment length of 150 bp and a minimum identity at 75%^40^. The hypervariable regions were filtered by using PH LANE mask (http://greengenes.lbl.gov/). FastTree 2.1 (http://www.microbesonline.org/fasttree/ ) was adopted for building the phylogenetic tree with Kimura’s 2-parameter model (https://www.megasoftware.net › mega4 › distance_models). The estimation of diversity indices and generation of rarefaction plots were completed in QIIME. A Venn diagram for each intestinal segment was generated based on the OTUs distributed among four emu samples. Comparison of SI microbiota diversity was done using SYSTAT 9 for Windows (SPSS Science, Chicago, Illinois) and pair-wise comparison was done with Wilcoxon Test.

### Microbial metabolic function prediction

The PICRUSt (phylogenetic investigation of communities by reconstruction of 185 unobserved states)^41^ was employed to predict functional genes of the classified members of the microbiome (including Cecal OTU data (SRE accession number SRA071216) obtained from Bennett et al.^13^ through closed-reference based OTU mapping against the Greengenes database^41^. Mapped closed-reference OTUs are normalized based on the copies of 16S rRNA gene within the known bacterial genomes in Integrated Microbial Genomes (IMG). Predicted genes were clustered hierarchically and categorized on the basis of KEGG^42^ orthologues (KO’s) and pathways (level -3). Significantly different pathways were identified by using STAMP software^43^. To compare differences in predicted metagenomic functions among ceca and different intestinal segments, Welch’s *t*-test was applied on the predicted microbiome functions determined by KEGG functional modules (level-3) under various microbiome metabolism^44^.

### Ethics approval

All experiments were performed in accordance with protocols reviewed and approved by the University of British Columbia Animal Care and Use Committee (Certificate # A10-0106).

## Results

### Richness of SI microbiota

After stringent quality filtering and trimming, a total of 165,585 sequencing reads (average 41,396 ± 3,266 seqs/bird) were generated from the 3 SI segments (duodenum, jejunum and ileum) in the 4 emus (2 males and 2 females). Average sequencing reads were 12,982 ± 1062 seqs/bird (See also Supplemental Table S1 and Supplemental Fig. S7).

**Figure 1:**
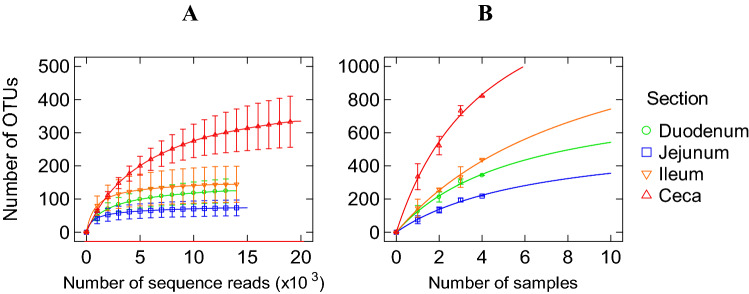
Rarefaction analysis, calculated at 97% dissimilarity, for the assessment of operational taxonomic unit (OTU) coverage within the16S rRNA gene–based bacterial communities in the gastrointestinal tract of the four emus (*Dromaius novaehollandiae*) sampled in this study. (**A**) The number of OTUs as a function of the number of sequence reads. (**B**) The number of OTUs as a function of the number of individual emu sampled.

The sequences were classified into 701 (average 262.8 ± 55.3/sample) species-level OTUs in the different SI segments. Only 2 OTUs (*Escherichia* and *Sinobacteraceae*) were identified as core bacteria along the small intestine segments and only *Escherichia* was core OTU of both small intestine and ceca^13^. The two OTUs (OTU_73 and OTU_773) were shared by four individuals among 8 samples (see Supplemental Table S3). Refraction curves (Fig. 1) showed that the curves for all 3 SI segments were much flatter than the curve for cecum^13^. Since the cecum curve indicated that we were capturing about 50% of possible cecal OTUs in the population^13^, the much flatter and approaching plateau curves for the 3 SI segments would indicate that we have captured most of the possible OTUs in the sampled population.

#### Duodenum

**Figure 2:**
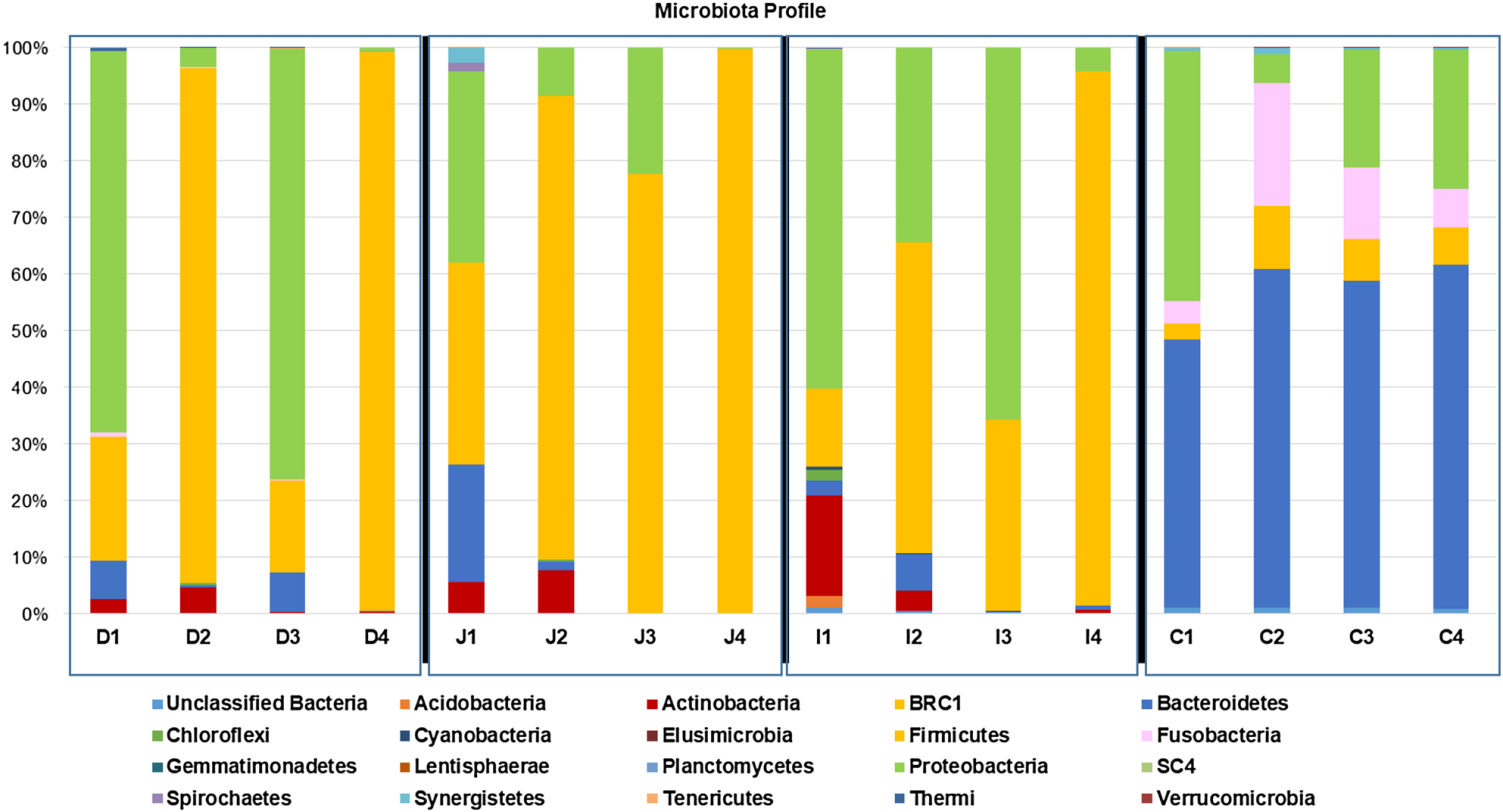
Phylum level microbial profile of the 4 intestinal compartments (D: duodenum, J: jejunum, I: ileum, C: ceca).

**Figure 3:**
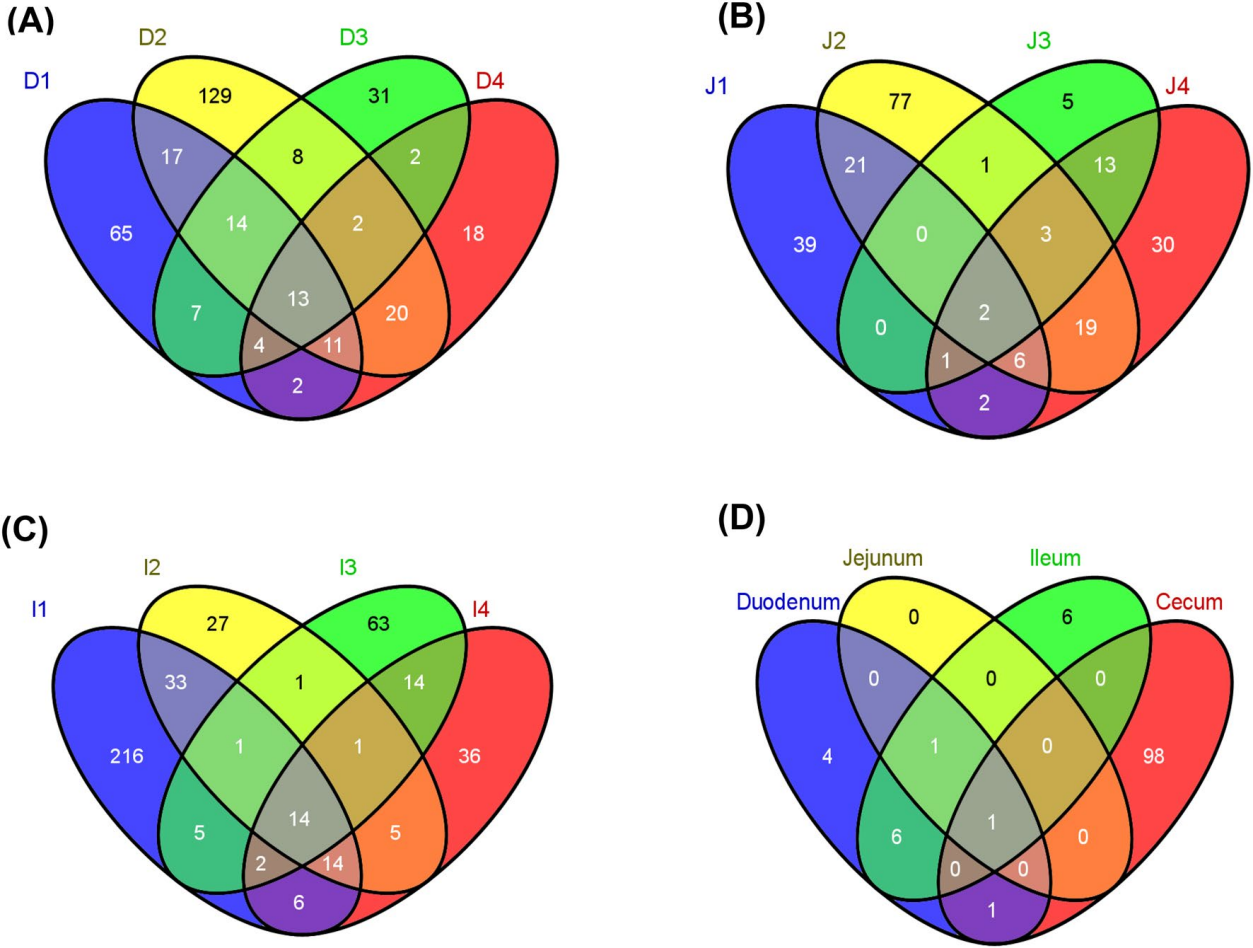
Venn diagram showing (**A**) the distribution of all 343 duodenum OTUs, calculated at 97% dissimilarity, identified in the 52,880 16S rDNA sequences. 13 OTUs were common to all 4 individuals. (**B**) The distribution of all 219 Jejunum OTUs identified in 61,139 sequences. 2 OTUs were common to the 4 individuals. (**C**) The distribution of all 438 ileum OTUs identified in 51,567 sequences. 14 OTUs were common to the 4 individuals. (**D**) The distribution of 129 OTUs common in all 4 individual in the different intestinal segments. Only 1 OUT was found in all 4 segments. The cecum data were obtained from Bennett et al.^13^ (D1–D4: duodenum of the 4 emus examined, J1–J4: jejunum, I1–I4: ileum, C1–C4: ceca).

The duodenum segment yielded a total of 52,880 sequences (13,220 ± 290 seqs/bird) (Table 1). The duodenum sequences were classified into 343 OTUs (125.0 ± 32.6 OTUs/bird) mainly belonging to 4 microbial phyla; *Firmicutes*, *Proteobacteria*, *Bacteroidetes*, and *Actinobacteria* (Fig. 2). In total, 13 OTUs, accounting for 74.1% of the sequence reads, were common to all 4 duodenal samples and 243 OTUs were unique to individual emus (Fig. 3A). Notably, *Turicibacter* (*Firmicutes*) accounted for 31.7% of total sequence reads (Table 2).

**Table 1:** The number of sequence reads and OTUs detected in 4 emus. Cecum data are obtained from Bennett et al.^13^

	Sequence reads		Number of OTU	
	Total	Mean ± SE	Total	Mean ± SE
Duodenum	165,585	13,220 ± 290	343	125.0 ± 32.6
Jejunum		15,285 ± 2,366	219	75.3 ± 21.3
Ileum		12,982 ± 1,062	438	145.3 ± 48.7
Cecum^1^	69,194	17,299 ± 2,113	821	335.0 ± 70.3

^1^Darin et al.^13^.

**Table 2:** Dominant OTUs found in duodenum and ileum.

	Duodenum	Ileum
Genus	% Sequence reads	% Sequence reads
*Turicibacter* (OTU748)	31.7	19.7
Unknown Proteobacteria (OTU512)	12.9	4.3
*Escherichia* (OTU73)	11.2	18.9
Unknown Clostridiaceae (OTU132)	8.5	6.3
*Lactobacillales* (OTU305)	5.7	6.1
*Streptococcus* (OTU430)	1.6	Not found
*Bacteroids* (OTU459)	1.1	Not found
*Sinobacteraceae* (OTU773)	0.5	1.0
Total	73.2	50.2

#### Jejunum

The jejunum segment yielded 61,139 sequences (15,285 ± 2,366 seqs/bird) (Table 1). The sequences were classified into the same 4 main phyla as in the duodenum (Fig. 2). In total, 2 OTUs, accounting for 11.2% of the sequence reads, were common in all 4 jejunal samples and 10 OTUs, accounting for 50.9%, were common in 3 emus. One hundred and fifty-one OTUs were unique to individual emus (Fig. 3B). In all 4 emus, *Escherichia* (Proteobacteria) and *Sinobacteraceae* (Proteobacteria) accounted for 6.6% and 4.6% of the sequence reads, respectively. *Lactobacillales* (19.8%), unknown *Clostridiaceae* (*Firmicutes*) (17.6%) and *Streptococcus* (*Firmicutes*) (10.1%) were found in at least 3 emus. *Turicibacter* was found in 2 emus (5.1%).

#### Ileum

The ileum segment yielded 51,567 sequences (12,892 ± 1,062 seqs/sample) (Table 1). The sequences were classified into the same 4 main phyla as in the previous 2 segments (Fig. 2). In total, 14 OTUs, accounting for 59.7% of the total sequence reads, were common to all 4 ileum samples and 342 OTUs were unique to individual emus (Fig. 3C). *Turicibacter* and *Escherichia* accounted for 19.7% and 18.9% of the total sequence reads, respectively (Table 2).

#### Ceca

Combining with data obtained from Bennett et al.^13^, the distribution of 129 OTUs common in all 4 individuals in the different intestinal segments was plotted in a Venn diagram (Fig. 3D). Only 1 OTU, *Escherichia* (*Proteobacteria*), was found in all 4 segments.

### A comparison of SI microbiota diversity

**Figure 4:**
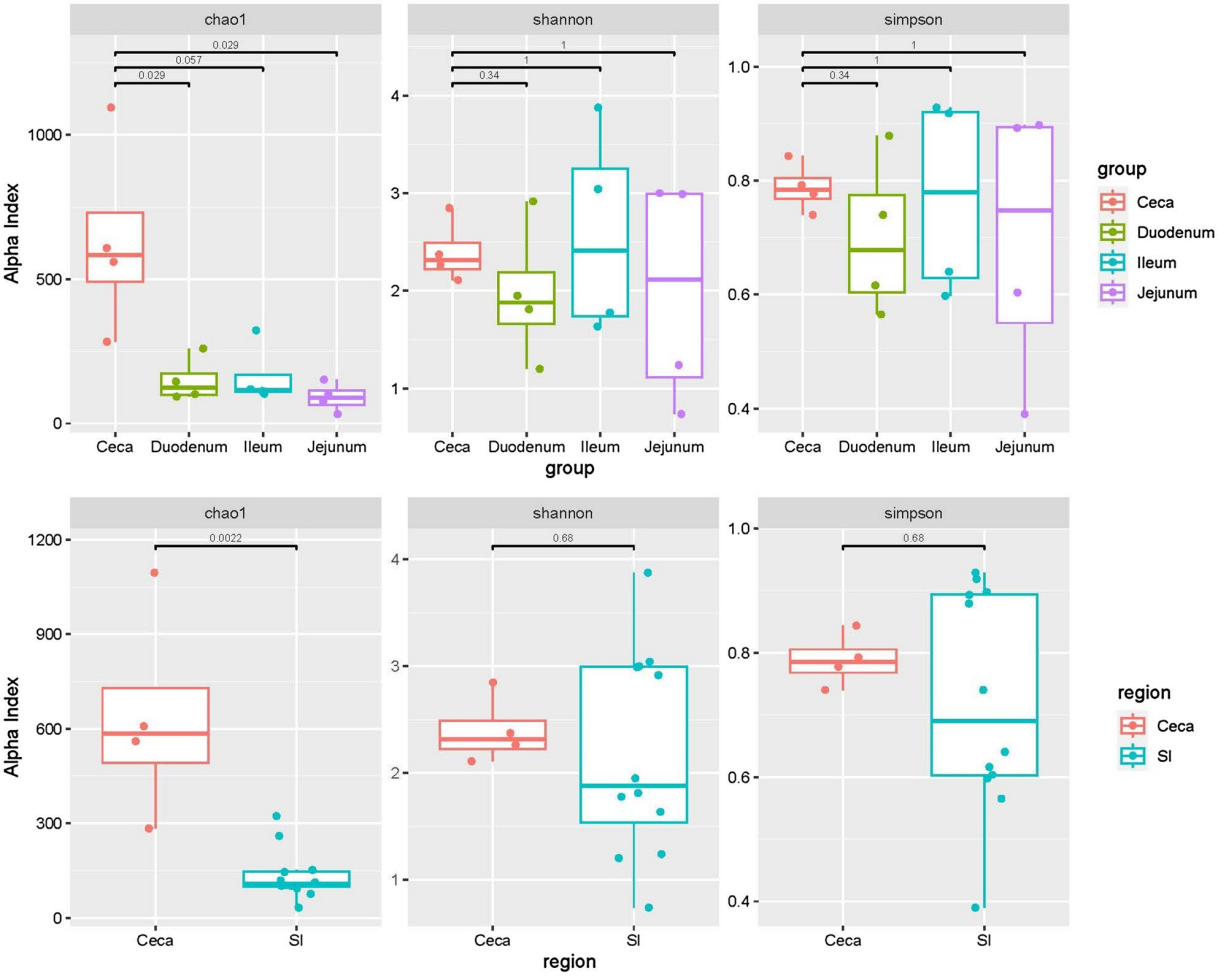
Pair-wise comparison of ceca microbiota species richness and diversity with SI microbiota species richness and diversity (D1–D4: duodenum of the 4 emus examined, J1–J4: jejunum, I1–I4: ileum, C1–C4: ceca).

The estimated microbial richness by Chao1 index of the duodenum, jejunum, and ileum was 150 ± 77 OTUs, 164 ± 106 OTUs, and 91 ± 50 OTUs, respectively. The estimated microbial diversity by Shannon index was 1.97 ± 0.71, 2.58 ± 1.1, and 1.99 ± 1.18, respectively; by Simpson index was 0.7 ± 0.14, 0.78 ± 0.18, and 0.7 ± 0.25, respectively (Table 3). The Ceca Chao1 index (624 ± 170) was significantly (*P* = 0.0011) higher than those of the SI segments, whether Ceca was compared with the whole SI or the three SI segments respectively (Ceca vs duodenum *P* = 0.01429; ceca vs ileum *P* = 0.0286; ceca vs jejunum *P* < 0.01429; by Wilcoxon Test) (Fig. 4). There was no significant difference in the Shannon and the Simpson indices among the 4 intestinal segments.

**Table 3:** Richness and diversity estimation for bacterial community, as indicated by Chao1, Shannon and Simpson indices, in 3 SI segments (duodenum, ileum and jejunum) of the 4 emus sampled.

Intestine		Species richness	Species diversity	
Compartment	Sample	Indices	Indices	
		Chao1	Shannon	Simpson
Duodenum	D1	145.36	2.92	0.88
	D2	259.92	1.95	0.62
	D3	93	1.81	0.74
	D4	101.55	1.2	0.57
	Mean ± SD	150 ± 77	1.97 ± 0.71	0.7 ± 0.14
Ileum	I1	322.89	3.88	0.93
	I2	118.75	3.04	0.92
	I3	112.67	1.78	0.64
	I4	102.91	1.63	0.6
	Mean ± SD	164 ± 106	2.58 ± 1.1	0.78 ± 0.18
Jejunum	J1	76	2.99	0.9
	J2	152	3	0.89
	J3	32.5	0.74	0.39
	J4	101.67	1.24	0.6
	Mean ± SD	91 ± 50	1.99 ± 1.18	0.7 ± 0.25
Cecum^1^	Mean ± SD	624 ± 170	3.40 ± 0.20	0.79 ± 0.02

^1^Darin et al.^13^.

### A comparison of SI and cecal microbiota between female and male emus

There were 7 OTUs that were found only in the cecal contents of all female emus (Table 4) and 8 OTUs that were found only in all male ceca (Table 5). The SI contents were more variable. There were 18 OTUs found only in the SI contents of all female emus (Table 6) and 59 OTUs in males (Table 7). There was not enough replicated samples for statistical comparison of male and female microbiota.

**Table 4:** OTUs found only in the cecal contents of all female emus.

Phylum	Class	Order	Family	Genus	Number of OTUs
Unclassified	**–**	**–**	**–**	**–**	1
*Proteobacteria*	*Gammaproteobacteria*	*Enterobacteriales*	*Enterobacteriaceae*	Unclassified	2
*Firmicutes*	*Clostridia*	*Clostridiales*	*Lachnospiraceae*	Unclassified	1
*Bacteroidetes*	*Bacteroidia*	*Bacteroidales*	*Bacteroidaceae*	*Bacteroides*	2
*Fusobacteria*	*Fusobacteria*	*Fusobacteriales*	*Fusobacteriaceae*	Unclassified	1
Total					7

**Table 5:** OTUs found only in the cecal contents of all male emus.

Phylum	Class	Order	Family	Genus	Number of OTUs
Unclassified	–	–	–	–	1
*Firmicutes*	*Clostridia*	*Clostridiales*	*Lachnospiraceae*	*Eubacterium*	1
				Unclassified	2
			*Ruminococcaceae*	Unclassified	3
*Actinobacteria*	*Actinobacteria*	*Coriobacteriales*	*Coriobacteriaceae*	*Eggerthella*	1
Total					8

**Table 6:** OTUs found only in the small intestinal contents of all female emus.

Phylum	Class	Order	Family	Genus	Number of OTUs
Unclassified	–	–	–	–	1
*Proteobacteria*	*Gammaproteobacteria*	*Enterobacteriales*	*Enterobacteriaceae*	*Escherichia*	1
				Unclassified	5
*Firmicutes*	*Bacilli*	*Lactobacillales*	*Streptococcaceae*	*Lactococcus*	1
				*Streptococcus*	1
				Unclassified	1
			Unclassified	–	4
	*Clostridia*	*Clostridiales*	*Clostridiaceae*	Unclassified	1
	Unclassified	–	–	–	1
*Actinobacteria*	*Actinobacteria*	*Actinomycetales*	*Micrococcaceae*	*Arthrobacter*	1
*Bacteroidetes*	*Bacteroidia*	*Bacteroidales*	*Prevotellaceae*	Unclassified	1
Total					18

**Table 7:** OTUs found only in the small intestinal contents of all male emus.

Phylum	Class	Order	Family	Genus	Number of OTUs
*Proteobacteria*	*Gammaproteobacteria*	*Xanthomonadales*	*Xanthomonadaceae*	*Lysobacter*	1
				Unclassified	1
		*Pseudomonadales*	*Moraxellaceae*	Acinetobacter*	1
				Unclassified	1
		*Alteromonadales*	*Alteromonadaceae*	*Alishewanella*	1
	*Alphaproteobacteria*	*Rhodobacterales*	*Rhodobacteraceae*	*Paracoccus*	3
				*Rhodobacter*	1
		*Rhizobiales*	*Phyllobacteriaceae*	*Mesorhizobium*	1
				*Devosia*	1
				*Bosea*	1
		*Sphingomonadales*	*Sphingomonadaceae*	*Sphingopyxis*	1
			Unclassified	–	1
	*Betaproteobacteria*	*Hydrogenophilales*	*Hydrogenophilaceae*	*Hydrogenophilus*	1
		*Burkholderiales*	*Oxalobacteraceae*	Unclassified	1
			Unclassified	–	1
Firmicutes	*Bacilli*	*Bacillales*	*Staphylococcaceae*	*Macrococcus*	1
				*Jeotgalicoccus*	1
			*Bacillaceae*	*Bacillus*	1
		*Lactobacillales*	*Streptococcaceae*	*Streptococcus*	1
			*Carnobacteriaceae*	*Trichococcus*	1
			*Aerococcaceae*	*Aerococcus*	1
		Unclassified	–	–	1
	*Clostridia*	*Clostridiales*	*Peptostreptococcaceae*	*Tepidibacter*	1
			*FamilyXI.IncertaeSedis*	Unclassified	1
			*Catabacteriaceae*	Unclassified	1
			*Clostridiaceae*	*Clostridium*	1
		Unclassified	–	–	1
*Actinobacteria*	*Actinobacteria*	*Actinomycetales*	*Micrococcaceae*	*Arthrobacter*	1
			*Corynebacteriaceae*	*Corynebacterium*	2
			*Nocardioidaceae*	*Marmoricola*	1
				*Nocardioides*	1
				Unclassified	1
			*Microbacteriaceae*	*Microbacterium*	1
			*Gordoniaceae*	*Gordonia*	1
			*Dietziaceae*	*Dietzia*	1
			*Nocardiaceae*	*Rhodococcus*	1
			*Propionibacteriaceae*	*Tessaracoccus*	1
			*Dermabacteraceae*	*Brachybacterium*	1
			*Brevibacteriaceae*	*Brevibacterium*	1
			Unclassified	–	2
		*Acidimicrobiales*	*Iamiaceae*	Unclassified	1
*Bacteroidetes*	*Sphingobacteria*	*Sphingobacteriales*	*Flexibacteraceae*	*Cytophaga*	1
			*Sphingobacteriaceae*	*Sphingobacterium*	2
				Unclassified	1
			Unclassified	–	2
	*Bacteroidia*	*Bacteroidales*	*Porphyromonadaceae*	Unclassified	1
	*Flavobacteria*	*Flavobacteriales*	*Flavobacteriaceae*	*Gelidibacter*	1
				*Flavobacterium*	1
				*Capnocytophaga*	1
				*Myroides*	1
*Chloroflexi*	*Thermomicrobia*	*HN1-15*	Unclassified	–	2
*Cyanobacteria*	*4C0d-2*	*mle1-12*	Unclassified	–	1
Total					59

*Also found in the cecal content of 1 male emu.

### A comparison of predicted microbial metabolic function

**Figure 5:**
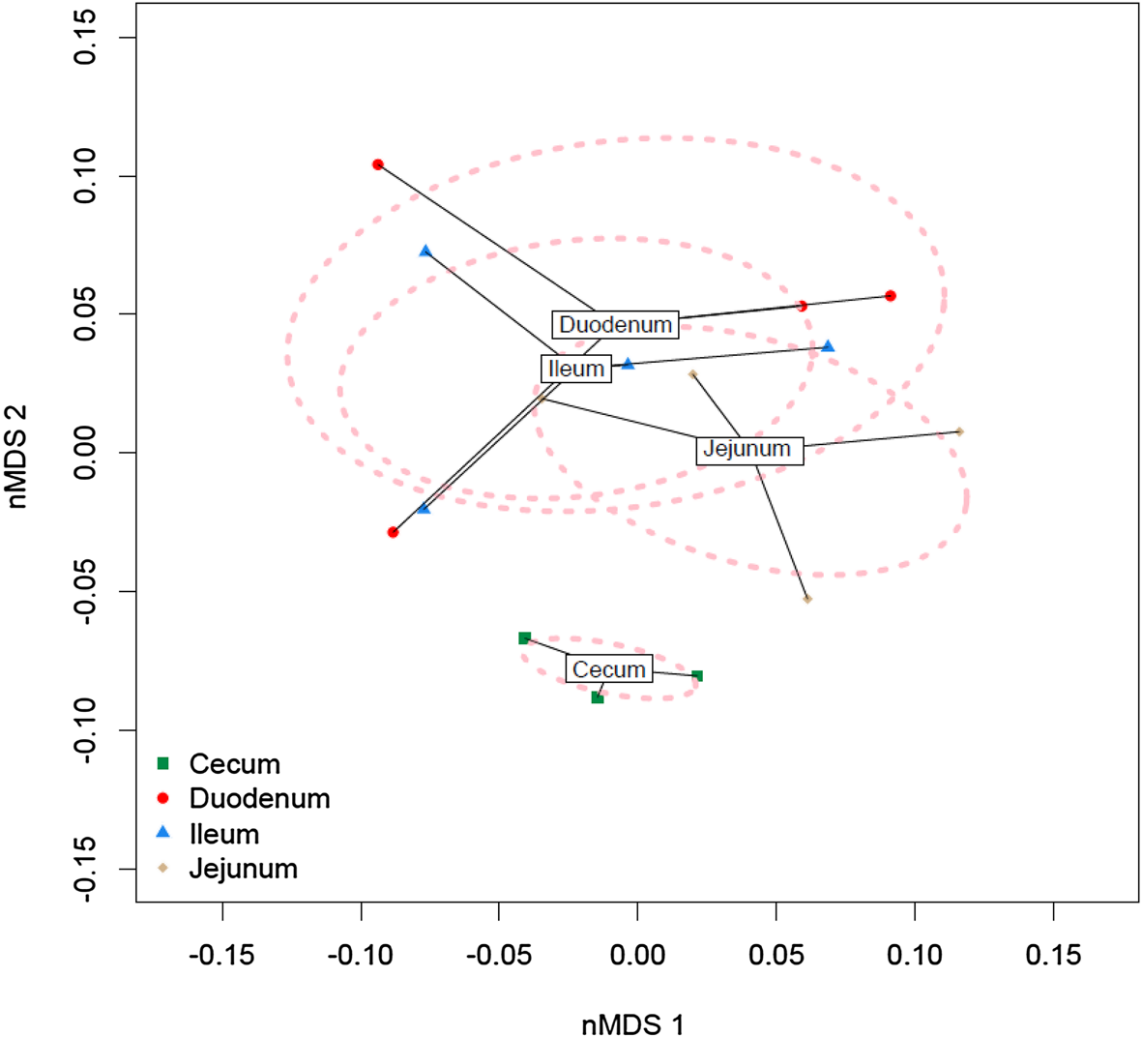
Two-dimensional non-parametric multidimensional scaling (NMDS) ordination plots of predicted bacterial KEGG pathways in cecal and small intestinal segments (Duodenum, jejunum and Ileum) samples of emus (*n* = 4 per gut site). Each dot represents an individual samples; the circles indicate the SD. The label box is the mean of each group.

PICRUSt nMDS plot (Fig. 4) showed that the predicted metagenomics functions of the cecum are very different from that of the small intestine. The size of the cecum cluster in the nMDS plot was much smaller than the clusters formed by samples from other intestinal segments and did not overlap with the other segment clusters. In the small intestine, Duodenum and Ileum have more similar microbiome metabolic functions compared to that of the jejunum. The 4 emus sampled were more uniform in the distribution of Ceca OTUs and more diverse in small intestine OTUs (Supplemental Fig. S1). Similarly, individual differences of microbiome functions in ceca are less than the small intestine segments (Fig. 5). (D1–D4: duodenum of the 4 emus examined, J1–J4: jejunum, I1–I4: ileum, C1–C4: ceca) .

The PICRUSt comparison of predicted microbiome metabolic functions among the cecum and the small intestinal compartment is shown in Supplemental Figs. S2 (Cecum vs Duodenum), S3 (Cecum vs Ileum), S4 (Cecum vs Jejunum), S5 (Duodenum vs Jejunum) and S6 (Ileum vs Jejunum). There was no significant difference between Duodenum and Ileum.

To summarize, microbiotic metabolic functions mostly in the duodenum are shown in Table 8. Microbiotic metabolic functions mostly in the Jejunum are shown in Table 9. Microbiotic metabolic functions more in the Ileum than the cecum are shown in Table 10. Microbiotic metabolic functions mostly or exclusively in the cecum are shown in Table 11.

**Table 8:** Significant differences in microbiota metabolic functions between the Duodenum and the other three intestine segments.

Duodenum Microbiota metabolic functions	> Jejunum	> Ileum	> Ceca
Metabolism of co-factors and vitamins			*P* < 0.004
D-alanine metabolism			*P* < 0.02
Riboflavin metabolism	*P* < 0.032		*P* < 0.025
Atrazine degradation			*P* < 0.03
Xylene degradation			*P* < 0.035

See also Figs. S2 and S5.

**Table 9:** Significant differences in microbiota metabolic functions between the Jejunum and the other three intestine segments.

Jejunum Microbiota metabolic functions	> Duodenum	> Ileum	> Ceca
D-alanine metabolism	*P* < 0.015	*P* < 0.027	*P* < 0.003
Biosynthesis of ansamycines (antibiotics)	*P* < 0.0045		
Phosphonate and phosphinate metabolism	*P* < 0.01		
Purine metabolism	*P* < 0.022	*P* < 0.028	
Xylene degradation			*P* < 0.006
Chloroalkane and chloroalkene degradation			*P* < 0.008
Styrene degradation			*P* < 0.0065
Dioxin degradation			*P* < 0.0072
Translation protein			*P* < 0.0076
Lipid metabolism			*P* < 0.0062
Fatty acid biosynthesis			*P* < 0.017
Butanoate metabolism			*P* < 0.018
Tyrosine metabolism			*P* < 0.019
Benzoate degradation			*P* < 0.020
Synthesis and degradation of ketone bodies			*P* < 0.029

See also Figs. S5 and S6.

**Table 10:** Significant differences in microbiota metabolic functions between the Ileum and the other three intestine segments.

Ileum Microbiota metabolic functions	> Duodenum	> Jejunum	> Ceca
Xylene degradation			*P* < 0.004
Metabolism of co-factors and vitamins			*P* < 0.0021
D-alanine metabolism			*P* < 0.0015
Glutathione metabolism			*P* < 0.015
Chloroalkane and chloroalkene degration			*P* < 0.024
Retinol degradation			*P* < 0.024
Drug metabolism – cytochrome P450			*P* < 0.026
Tyrosine metabolism			*P* < 0.033
Dioxin degradation			*P* < 0.033

See also Fig. S3.

**Table 11:** Significant differences in microbiota metabolic functions between the ceca and the three small intestine segments.

Ceca Microbiota metabolic functions	> Duodenum	> Jejunum	> Ileum
Protein digestion and absorption	*P* < 0.002	*P* < 0.00016	*P* < 0.0001
Steroid hormone biosynthesis	*P* < 0.0006	*P* < 0.0009	*P* < 0.0003
Insulin signaling pathway	*P* < 0.049	*P* < 0.00007	*P* < 0.0008
Amino sugar and nucleotide sugar metabolism	*P* < 0.002		*P* < 0.002
Glycosphingolipid biosynthesis—globo series	*P* < 0.0008	*P* < 0.0006	*P* < 0.002
Glycosphingolipid biosynthesis—ganglio series	*P* < 0.0005	*P* < 0.0008	*P* < 0.002
Other Glycan degradation	*P* < 0.0006	*P* < 0.0013	*P* < 0.001
Glycosaminoglycan degradation	*P* < 0.0007	*P* < 0.0017	*P* < 0.001
Sphingolipid metabolism	*P* < 0.0015	*P* < 0.0015	*P* < 0.002
Alanine, aspartate and glutamate metabolism	*P* < 0.001	*P* < 0.0085	*P* < 0.003
Galactose metabolism	*P* < 0.009		*P* < 0.004
Glutametergic synapse	*P* < 0.001	*P* < 0.046	*P* < 0.028
Restriction enzyme	*P* < 0.001	*P* < 0.001	*P* < 0.001
Nitrogen metabolism		*P* < 0.0021	
Other ion-coupled transporters		*P* < 0.0004	
Carbohydrate digestion and absorption	*P* < 0.030	*P* < 0.0004	*P* < 0.007
Purine metabolism	*P* < 0.006		*P* < 0.004
Zeatin biosynthesis	*P* < 0.011	*P* < 0.010	*P* < 0.009
Biotin metabolism	*P* < 0.026	*P* < 0.010	*P* < 0.003
Streptomycin biosynthesis	*P* < 0.005	*P* < 0.014	*P* < 0.010
Sulfur metabolism		*P* < 0.032	*P* < 0.011
Polyketide sugar unit biosynthesis	*P* < 0.017	*P* < 0.009	*P* < 0.011
Carbon fixation in photosynthetic organisms		*P* < 0.023	*P* < 0.011
Phenylpropanoid biosynthesis		*P* < 0.012	*P* < 0.042
Antigen processing and presentation	*P* < 0.046	*P* < 0.0019	*P* < 0.012
NOD-like receptor signaling pathway	*P* < 0.046	*P* < 0.0021	*P* < 0.012
Energy metabolism		*P* < 0.026	*P* < 0.013
Butirosin and neomycin biosynthesis	*P* < 0.017		*P* < 0.015
Secondary bile acid biosynthesis	*P* < 0.042		*P* < 0.017
Phosphonate and phosphinate metabolism	*P* < 0.0026		*P* < 0.02
Vitamin B6 metabolism	*P* < 0.037	*P* < 0.005	*P* < 0.021
Other transporters	*P* < 0.049	*P* < 0.0027	*P* < 0.024
Nitrogen metabolism		*P* < 0.0021	
Biosynthesis of vancomycin group antibiotics	*P* < 0.0026	*P* < 0.0024	*P* < 0.0034
Adipocytokine signaling pathway		*P* < 0.0049	*P* < 0.044
Lipopolysaccharide biosynthesis protein		*P* < 0.016	*P* < 0.040
Methane metabolism	*P* < 0.013		
Isoquinoline alkaloid biosynthesis	*P* < 0.023		*P* < 0.020

See also Figs. S2, S3, and S4.

## Discussion

Gut microbiota can be considered as an additional organ due to its vital importance on the physiological, metabolic, immunological, and digestion and nutritional uptake functions of the host^45^. Gastrointestinal microbiota may include bacteria, protists, yeasts, archaea, and viruses/phages. However, due mostly to their abundance and diversity, and limitations in sequencing techniques, a bacterial focus dominates the research field^46^. Birds host complex gastrointestinal bacterial communities that facilitate their biological roles, distribution, and diversity. However, the gut microbiota of birds has been poorly studied, especially in wild species under natural conditions. Studies of avian gut microbiota are dominated by research on domestic poultry^47^. Poultry are unlikely to be representative of all bird species. Birds are diverse and vary in life-history traits such as migratory behavior, flight capacity, diet, mating systems, longevity and physiology, all of which may impact gut microbiota. However, most microbes recovered from birds show little evidence of host specificity. Among the bird species, the flightless orders (ostriches, emus, cassowaries, kiwis, and rheas), and the weak fliers from the related Tinamiformes (tinamous) were among those hosting communities with median specificities approaching those of mammals. The microbiota of these bird orders (constituting the Palaeognathae) were also more likely to occur in mammals^48^.

### Composition and distribution of emu GIT microbiota

Most studies of avian species have focused on characterizing the microbiota in the ceca due to its large bacterial diversity^13–16^. However, a few poultry studies have sampled multiple gut regions of the small intestine (SI) which are less rich and diverse than cecal samples^49–51^. These studies have found differences between the microbial communities across the SI and concluded that a sample taken from one site should not be taken as representative of the small intestine as a whole. Wilkinson, et al.^33^ found nine phyla of bacteria in the quail gastrointestinal tract (GIT); however, their distribution varied significantly among GIT sections. Our study found that the overall composition of the emu gut microbiota appears similar to the microbiota of other migratory birds^27,52^. The emu microbiota exhibits significant regionalization among gut regions and the SI microbiota richness and diversity also was significantly less than the cecum. On the other hand, similar to Sage Grouse^27^, the emu cecum samples displayed lower variability than samples from SI segments. The size of the cecum cluster in the NMDA plot (Fig. 5) was much smaller than the clusters formed by samples from SI.

Similar to most avian species, the emu gut microbiota were dominated by *Firmicutes* and *Proteobacteria*, with lower abundance of *Bacteroidetes* and *Actinobacteria*. In broiler chickens, several studies showed that *Lactobacilli* are the major bacterial population in three segments (duodenum, jejunum, and ileum) of the small intestine, whereas *Clostridium* and *Bacteroides* are dominant in the ceca^24,53^. In emu, the distribution of these phyla were also different in the different intestinal segments. *Firmicutes* being most dominant in the jejunum, sharing its dominance with *Proteobacteria* in the duodenum and ileum and with lowest abundance in the ceca. *Proteobacteria* were evenly distributed among the intestinal segments but had huge variations among individual birds ranging from 76% to 0.18%. *Bacteroidetes* was most abundant in the ceca and very low abundance in the SI segments. *Actinobacteria* has low abundance in the SI segments and almost absent in the ceca. A fifth phylum, *Fusobacteria*, was detected in the emu ceca with fair abundance but not detected in the SI segments.

*Firmicutes* plays a key role in degradation of fiber into volatile fatty acids that provide energy for the hosts^54^. An abundance of *Firmicutes* has been linked to obesity in humans^55^, and to weight gain in chickens^56^. There are no studies addressing *Firmicutes* function in wild birds^57^, but the positive relationship between *Firmicutes* abundance and mass gain and immune function in domestic chickens^58,59^ suggests similar roles of *Firmicutes* between mammals and birds^47^.

*Proteobacteria* are able to grow on a range of organic compounds including protein, carbohydrates, and lipids. Recent studies of the human gut microbiome have shown that despite their relatively lower abundance, *Proteobacteria* contribute to much of the functional variation^60^. Within the avian digestive tract, Proteobacterial function remains undetermined^61^. Never the less, metagenomic sequence data from cats and dogs show that *Proteobacteria* encode a number of functions that relate to their ability to grow aerobically such as respiration, utilization of propionate as a carbon source, and repair of protein from oxidative damage^62^. As such, it is postulated that the *Proteobacteria* contribute to homeostasis of the anaerobic environment of the GIT, and hence, the stability of the strictly anaerobic microbiota^62^. Among the Proteobacterial classes, *α-Proteobacteria* are abundant in wild birds (45%), in contrast to only 15% relative abundance in mammalian hosts^63^. In our study, at the phylum level, about 30% of the emu microbiota were *Proteobacteria* and they were evenly distributed among the different intestinal segments. However, in chickens, the ceca harbour a microbiota dominated by carbohydrate metabolism with a much lower occurrence of respirational genes^64^. Birds have fewer obligate anaerobes and more facultative anaerobes than mammals, but flightless birds have more obligate anaerobes than flighted birds as a proportion of their gut microbial communities^48^. Moreover, large flightless birds harbor more homogeneous microbiomes than smaller passerines^52^.

*Bacteroidetes* generally produce butyrate, an end-product of fermentation which is thought to have antineoplastic properties and thus plays a role in maintaining a healthy gut^65^. In emu, *Bacteroidetes* are found most abundant in ceca (Fig. 2)^13^. High abundance of *Bacteroidetes* is also found in the ceca of Japanese quail (*Coturnix japonica*) and ostrich (*Struthio camelus*)^14,66,67^, and may support the hypothesis that *Bacteroidetes* play a specific role in break-down of cellulose and other plant materials^68^.

*Actinobacteria* are the fourth most abundant phylum of microbes in the wild bird GIT, but no studies have investigated the function of *Actinobacteria* in wild or domestic birds^47^. In human, *Actinobacteria* are pivotal in the maintenance of gut homeostasis^69^. *Actinobacteria*, in particular *Bifidobacteria*, are involved in the biodegradation of resistant plant-derived carbohydrate starch^70^. Moreover, *Bifidobacteria* are suspected to be involved in the transformation of linoleic acid (LA) into conjugated linoleic acids (CLA) which enhance immune functions^71^. *Bifidobacteria* are able to produce large quantities of acetate (SCFA), which is crucial for providing energy to gut barrier epithelial cells turnover and for their potent antibacterial activity^72^. *Actinobacteria* were not detected in the ostrich ceca^14^. While not detected in the emu ceca^13^, they were found in low abundance in the SI segments in our study. Both the ostrich and the emu are flightless ratites that are ranged outdoor. In chickens, the access to range enriched *Bifidobacteria* in both the ileum and ceca^73^. However, our emu samples were obtained in November when the birds started to breed and restricted their feed intake. Fasting may affect the composition of microbiota in the ceca^74^.

In human, *Fusobacteria* are often studied in the context of pathogenicity. A rich community of *Fusobacteria* was frequently reported in the guts of carnivorous and omnivorous birds^75^. Up to one third of the vulture gut microbiota, and over half of the penguin microbiota, can consist of *Fusobacteria*^76–79^. Analyses of the vulture microbiota have revealed abundant populations of *Fusobacteria* that appear to be beneficial to the host bird^78^. *Fusobacteria* are also observed at a lower abundance in other carnivorous seabirds and the omnivorous bustard^80,81^. We have found *Fusobacteria* only in the emu ceca and not in the SI. Emus are mainly herbivorous and only opportunistic in catching insects and small rodents when plants and fruits are not available. Olsen^82^ also found *Fusobacteria* in the Greylag geese’ gut microbiome but this species was considered herbivorous, consuming a diversity of foods that includes leaves, roots, and seeds. *Fusobacteria* may be more common and play a more important role in the avian gut microbiota than previously thought. Interestingly, our sampling of emu gut microbiota was at the beginning of their breeding season and some of the individuals may have started reducing their feed intake already.

In human and in laboratory mice, males and females were observed to be characterized by a different microbiota community composition^83–85^. Sex had a significant effect on Japanese quail (*C. japonica*) ileum microbial community^86–88^. The male chicken's cecal microbiota indicated a closer relation with glycan metabolism, while in the female chickens it was more related with lipid metabolism^89,90^. In human^83,91^, mice^92^, Japanese quail^87^ and chickens^89^, sex difference of gut microbiota can also be affected by diet. Sex difference of gut microbiota in free-living birds has rarely been investigated^93^. Liu et al.^94^ has documented sex difference of gut microbiota in wild Great Bustards (*Otis tarda*). In our study, sex seems to affect the emu SI more so than the ceca. Similar to zebrafish, emu females showed higher abundance of intestinal *Proteobacteria* than males^95^. Because of the small sample size, our results should be interpreted with caution and need confirmation in future studies. Given that emus have reversed sex role in incubation and brooding, it would be worthwhile to further study the sex difference of gut microbiota in emus.

### Microbial metabolic functions

Identifying the microbial diversity of the host gut is a necessary first step, but provides only limited information on functional aspects of the microbial community^47^. It is not only important to understand what microbial species may be present in the intestine, but it is more critical to understand what physiological and metabolic processes are taking place taking the whole microbiome into account.

The avian gut microbiota, and specifically microbiota associated with the crop and ceca, may be involved in detoxification of plant materials and other food compounds. Phenols, resins, and saponins, plant defense compounds against herbivory, are usually associated with plant defenses against herbivory and are usually indigestible or toxic to birds but common in diets of herbivorous birds. The crop is the first region of the gut to process consumed food and is therefore a logical reservoir for detoxifying bacteria^3^. Emus are predominantly herbivorous but have no prominent crop. The detoxification of plant material and environmental chemicals seem to be performed by SI microbiota, especially those in the jejunum. PICRUSt comparisons indicated that the emu jejunum microbiome has more genes for [chloroalkane and chloroalkene degradation]—from insecticides and a/the toxic component of plants, [styrene degradation]—from plant source, [dioxin degradation]—from environmental contaminants, [xylene degradation]—from plant source, and [benzoate degradation]—from a plant source, than the ceca and other SI segments. The emu SI microbiome also has genes for [atrazine degradation] (duodenum)—herbicide; [D-alanine metabolism] to modulate pathogenic bacterial colonization and host defence (jejunum)^96^; [Retinol degradation] to regulate protective or pathogenic immune responses in the intestine to prevent colonization by enteric pathogens (ileum)^97^; [Biosynthesis of ansamycines] (jejunum)—antibiotics]; [Drug metabolism] (ileum)—cytochrome P450 to alter the metabolic outcome of environmental toxicants and heavy metals^98^. Emus, being free ranged outdoors, would be exposed to more environmental factors associated with foraging and would need stronger detoxification of ingested food and better protective or immune response to enteric pathogens than chickens which are kept indoors and fed a processed diet. The importance of maintaining a stable cecal environment for the commensal bacteria is further evidenced by the predicted metabolic functions of the emu cecal microbiota. PICRUSt comparisons indicated that the emu cecal microbiome has more genes than SI segments for [Glycosphingolipid biosynthesis—globo series], which play a role as receptors in pathogen invasion and also participate in the mechanism of resistance to *E. coli* F18^99^; [Zeatin biosynthesis] to induce resistance against pathogen infections^100^; [Streptomycin biosynthesis]—Streptomycin enhances commensal *E. coli* and kills competing bacteria^101^; [Polyketide sugar unit biosynthesis] which has antibacterial, antifungal, antiviral, immune-suppressing, and anti-inflammatory activity^102^; [Butirosin and neomycin biosynthesis], and [Biosynthesis of vancomycin group antibiotics]—Antibiotics; [Isoquinoline alkaloid biosynthesis]—which has antiviral, antibacterial, and antifungal functions^103^. Gut microbes can combat microbial pathogens directly through competitive exclusion^104^ or indirectly by activating the host immune system^105^. The emu cecal microbiome has more genes than the SI segments for [Antigen processing and presentation]—protein antigen is ingested, partially digested into peptide fragments, and then displayed for recognition by certain lymphocytes such as T cells^106^; [Lipopolysaccharide biosynthesis protein ]—gut microbial Lipopolysaccharide is thought to be one of the most potent activators of innate immune signaling and an important mediator of the microbiome’s influence on host physiology^107^; [Secondary bile acid biosynthesis]—A number of molecules either produced (e.g., volatile fatty acids) or transformed (e.g., trimethylamine, secondary bile acids) by gut microbiota is known to operate as signals in the host-microbe interplay and through their cognate receptors influence host metabolism and immunity to prevent gut dysbiosis^108^. The considerably longer retention time of digesta in the cecum, relative to other gut regions, also permits cecal microbial communities to stabilize and is likely the cause for the reduced variability observed among individuals^109^.

In chickens, the SI is the site for most digestion and practically all absorption of nutrients^109^. Sklan et al.^110^ reported that 95% of the fat was digested in the duodenum. Starch is the main carbohydrate in poultry feed. Starch granules are digested by pancreatic alpha-amylase in the SI^111^ and are absorbed by active transport. Duodenal microbiota is significantly associated with energy utilization^112^. It has been demonstrated that absorption of digestion products from fat, starch, and protein^113^ is to a large extent completed by the end of the jejunum^109^. The chicken ileum is mainly thought to play a role as a site for water and mineral absorption. It has been shown, however, that it may play a significant role in the digestion and absorption of starch in fast-growing broiler chickens^109,114^. Most of the undigested protein and fiber would undergo fermentation in the ceca and large intestine^115,116^.

In emus, microbial digestion and fermentation are mostly in the jejunum and ceca. There were significantly more jejunal microbiome genes than the other SI segments and the ceca for.

[Lipid metabolism]; [Fatty acid biosynthesis]—Short-chain fatty acids (SCFAs), are the main metabolites produced by bacterial fermentation of dietary fibers and resistant starch; [Butanoate metabolism]—The conversion of acetate to other SCFA, such as butyrate may be another regulatory approach for fat absorption and deposition^117^; [Synthesis and degradation of ketone bodies]—During fasting, a microbiota-dependent, Pparα-regulated increase in hepatic ketogenesis occurs, and myocardial metabolism is directed to ketone body utilization^118^. The samples were collected at the beginning of the breeding season when some of the individuals may have started fasting; [Tyrosine metabolism] (Jejunum, Ileum)—Bacterial tyrosine decarboxylases efficiently convert levodopa to dopamine. The abundance of bacterial tyrosine decarboxylases at the site of levodopa absorption in the SI had a significant impact on levels of levodopa in the plasma of rats^119^.

Herd & Dawson^120^ commented that the emu ileum may be where most of the fibre fermentation is taking place because the ileum had the largest amount of digesta and the emu ceca were small in comparison to other birds that used the ceca and colon for fibre fermentation and digestion. *Bacteroidetes* generally produce butyrate, an end product of fermentation that is thought to play a role in maintaining a healthy gut^65^. In emu, *Bacteroidetes* are found most abundant in the ceca. *Actinobacteria*, in particular *Bifidobacteria*, are involved in the biodegradation of resistant plant-derived carbohydrate starch, and *Actinobacteria* is more abundant in the emu ileum than other segments of the SI and not detectable in the ceca. *Turicibacter* has been correlated with butyrate and the consumption of a highly resistant starch diet in rats^121^. When pigs were fed with a high fibre diet, there was a significant increase in *Tericibacter* abundance in the ileal lumen^122^. In emu, *Tericibacter* were 32% and 20% of the sequence reads in the duodenum and ileum, respectively. In our study, the microbiome in the emu ceca had more genes for fermentation, digestion, and absorption than that of the ileum: [Carbohydrate digestion and absorption]; [Protein digestion and absorption]; [Energy metabolism]; [Nitrogen metabolism]; [Purine metabolism]; [Amino sugar and nucleotide sugar metabolism]; [Galactose metabolism]; [Methane metabolism]—Methane-producing microorganisms can improve fermentation efficiency by consuming any excess hydrogen and formate in the bowel, which subsequently improves acetate production and allows the body to absorb more nutrients and calories^123^; [Carbon fixation]—Gas is an inevitable product of microbial fermentation in the alimentary tract. The majority of bacterially generated gas comprises hydrogen, carbon dioxide, and methane. Gut bacteria can combine carbon dioxide with hydrogen to form carbohydrates (acetogenesis) for further digestion^124^. In the wild, emus feed largely on succulent herbage, seeds, fruits, flowers, and insects^125^ and it has been considered to make little use of microbial digestion in early studies^126^. However, later investigations into the dietary energy and nitrogen requirements indicated that the emus have appreciable digestion of plant fibre^127^. Emus were able to digest up to 45% of the fibre in their diets. *Firmicutes* plays a key role in the degradation of fiber into volatile fatty acids that provide energy for the hosts^54^. In our study, *Firmicutes* was most dominant in the jejunum, sharing its dominance with *Proteobacteria* in the duodenum.

We have fulfilled our objective to characterize the intraluminal intestinal bacterial community in the different SI segments. However, our samples were collected at the beginning of the breeding season and was a single time point sampling. In order to approach our long term goal of manipulating gut microbiota for improving emu fat production^128^, future research should explore seasonal variation of gut microbiome with association to reproductive state, changing diets and fat deposition^74^, age and sex variation, microbiota associated to the intestinal mucosa^129^, and the epigenetics of the intestinal mucosa^130,131^.

## Conclusion

The objective of this study was to characterize the intraluminal intestinal bacterial community in the different SI segments using pyrotag sequencing and compare that with the ceca. We found that the detoxification of plant material and environmental chemicals seem to be performed by SI microbiota, especially those in the jejunum. The emu cecal microbiome has more microbial genes than SI segments involving in protective or immune response to enteric pathogens. Microbial digestion and fermentation was mostly in the jejunum and ceca. This is the first study to characterize the microbiota of different compartments of the emu intestines via gut samples and not fecal samples. Results from this study allow us to further investigate the influence of the seasonal and physiological changes of intestinal microbiota on the nutrition of emus and indirectly influence the fatty acid composition of emu fat.

## Supplementary Information

Supplementary Information 1.

Supplementary Information 2.

Supplementary Information 3.

Supplementary Information 4.

## Data Availability

Cecal Tag-encoded pyrosequence data were deposited into NCBI Sequence Read Archive under accession number SRA071216.
