# Supplementary material for: Microbial diversity and metabolic function in duodenum, jejunum and ileum of emu (Dromaius novaehollandiae)
Source: Sci Rep. 2023 Mar 18;13:4488. doi: 10.1038/s41598-023-31684-8 (PMC10024708; doi:10.1038/s41598-023-31684-8)

**Supplemental Figure S1. UPGMA clustering calculated from (A) Jackknife-weighted and (B) Jackknife-unweighted UniFrac distance matrix based on OTU profiles, showing that the microbiota profiles of 4 emus sampled are more uniform in Cecum and more diverse in small intestine (duodenum, jejunum, and ileum). The colour of internal nodes represents the bootstrap values, with red for 75-100% support and yellow for 50-75%. C = cecal samples, I = ileial samples, J = Jejunal samples, D = duodenal samples.**


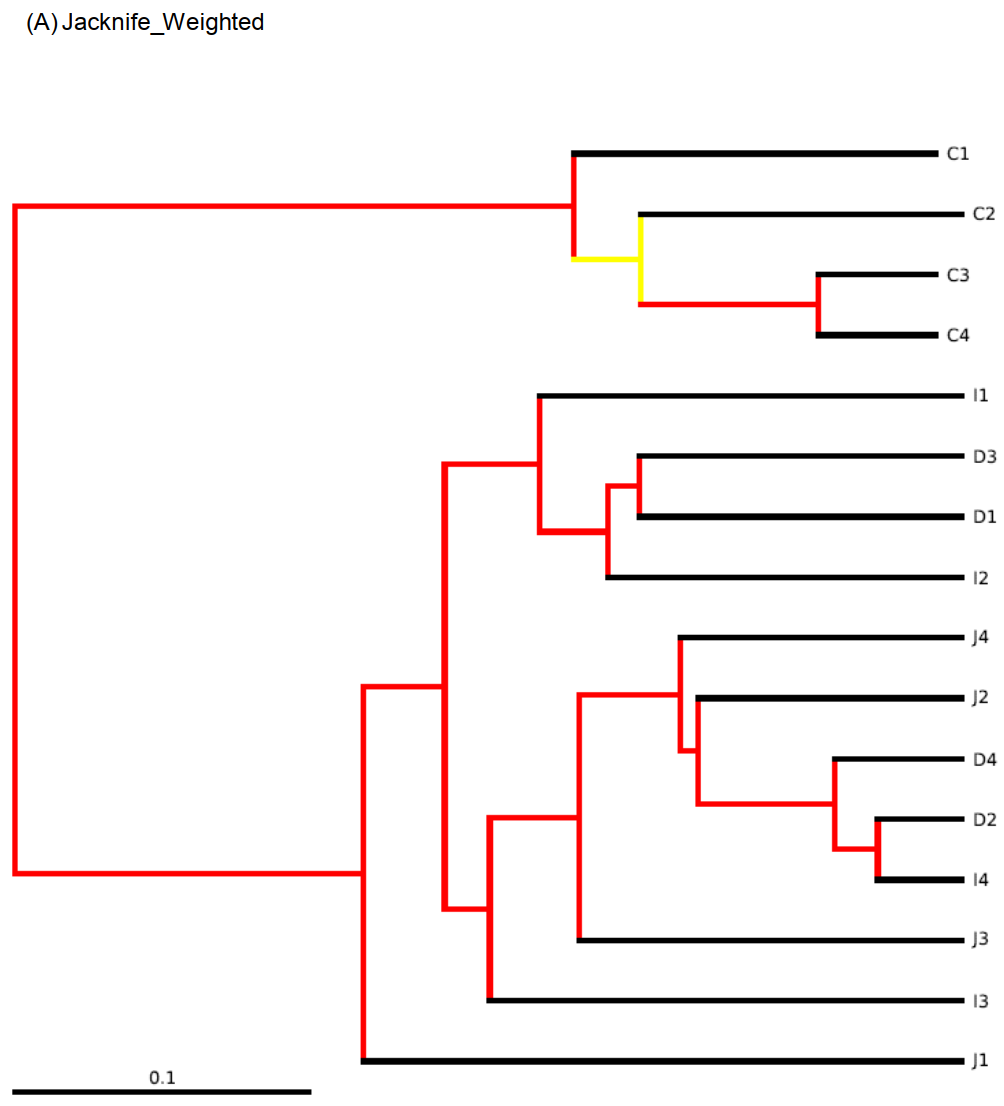

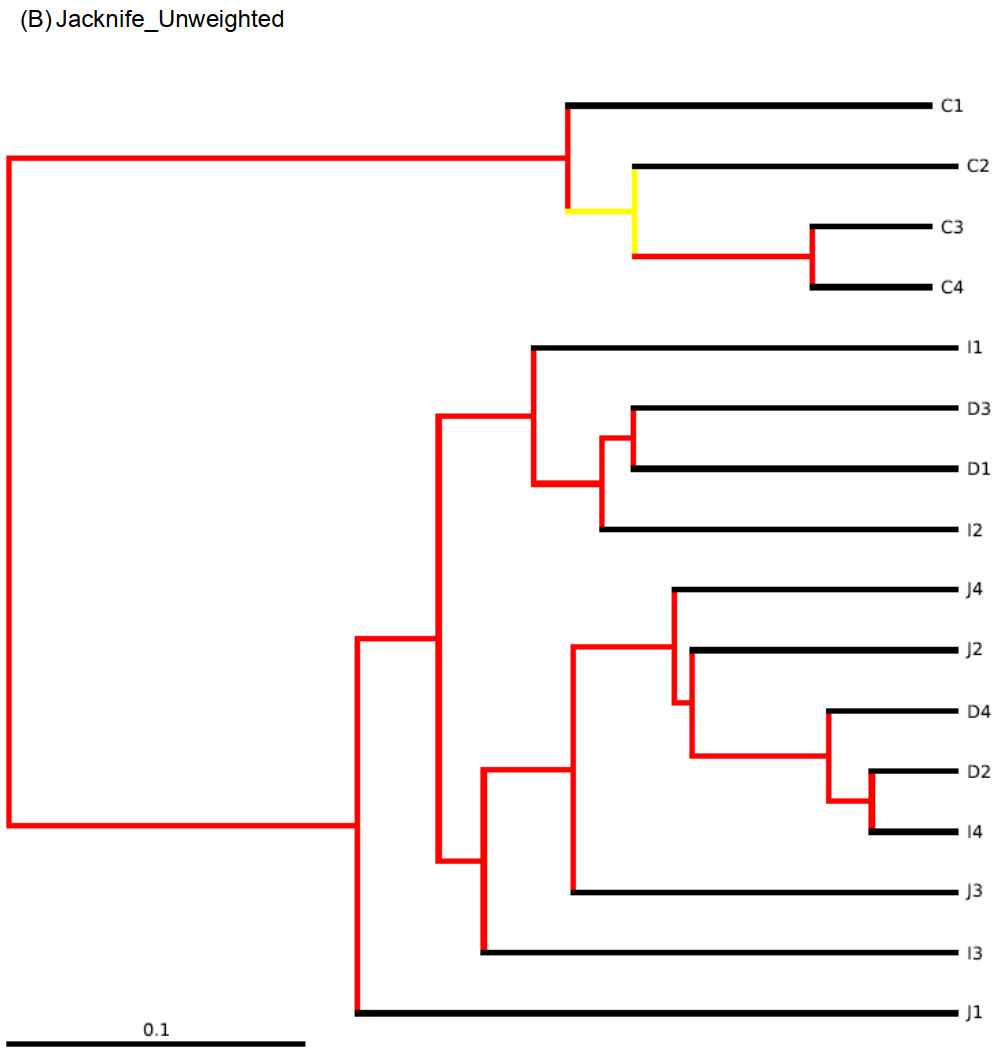


**Supplemental Figure S2 Comparison of predicted metagenomic functions between cecum and duodenum [41]. Only pathways with adjusted *P* value < 0.05 were displayed.**

**
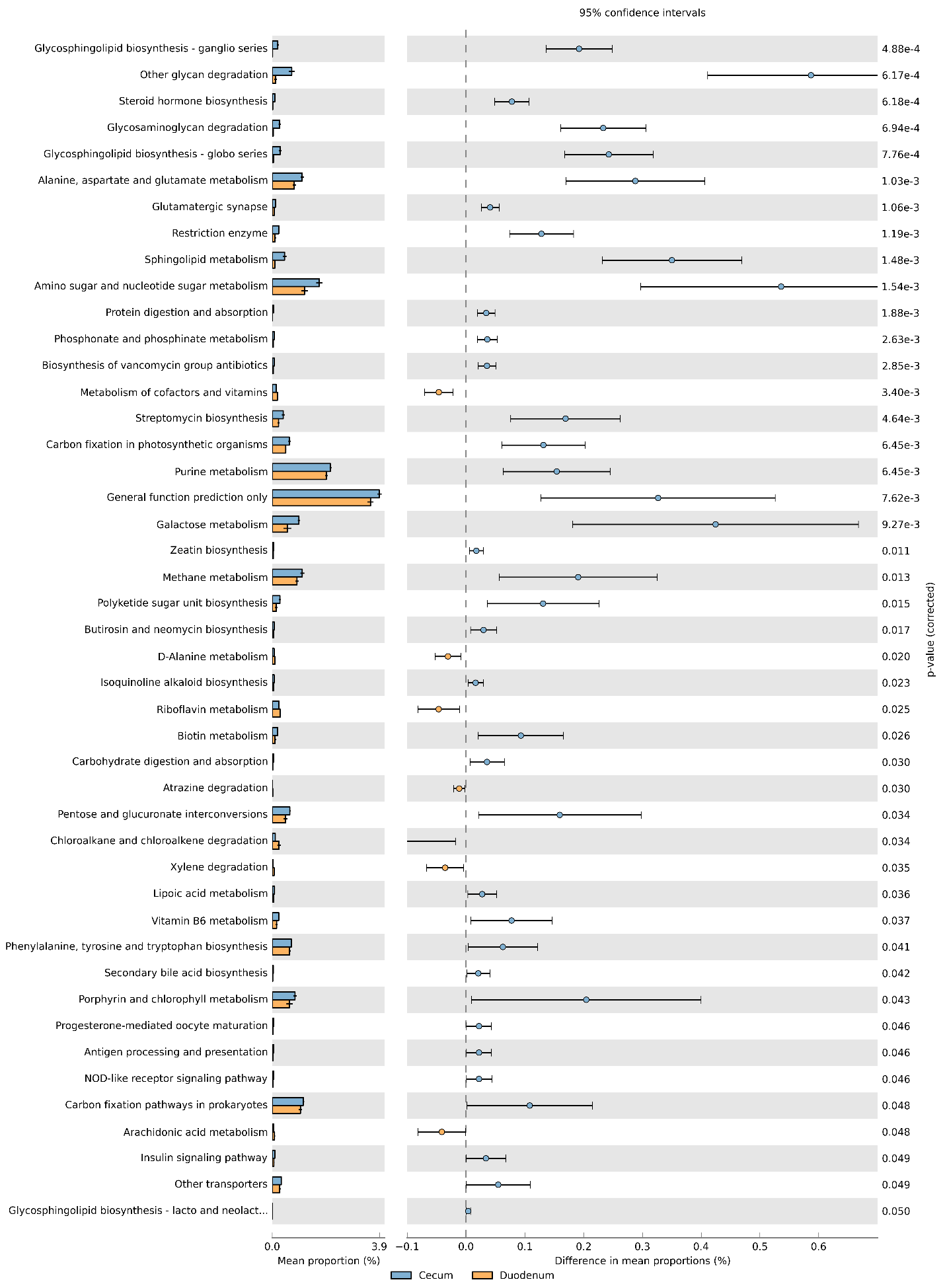
**

**Supplemental Figure S3 Comparison of predicted metagenomic functions between cecum and ileum [41]. Only pathways with adjusted *P* value < 0.05 were displayed.**





**Supplemental Figure S4 Comparison of predicted metagenomic functions between cecum and jejunum[41]. Only pathways with adjusted *P* value < 0.05 were displayed.**





**Supplemental Figure S5 Comparison of predicted metagenomic functions between duodenum and jejunum[41]. Only pathways with adjusted *P* value < 0.05 were displayed.**


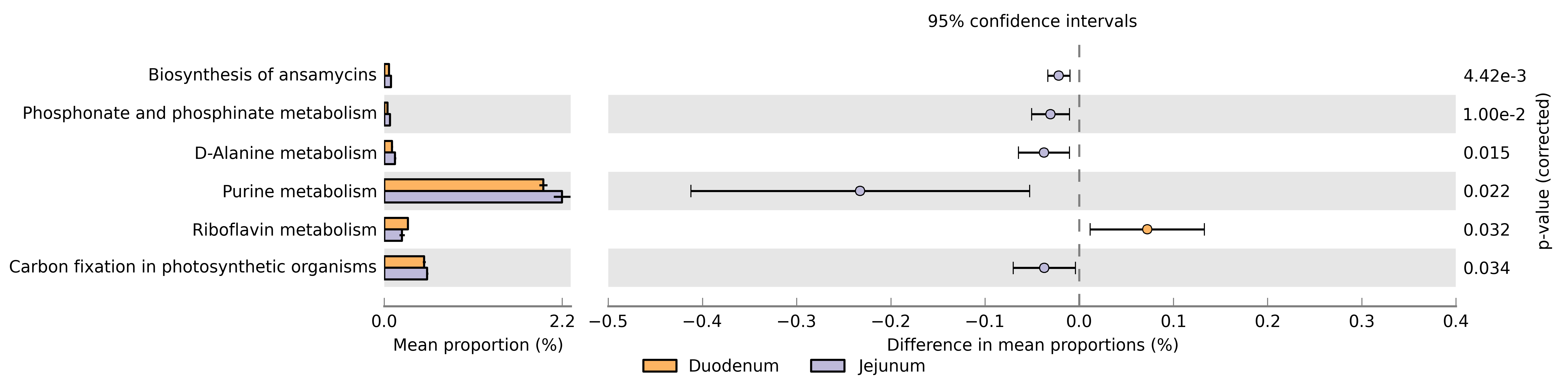


**Supplemental Figure S6 Comparison of predicted metagenomic functions between ileum and jejunum[42]. Only pathways with adjusted *P* value < 0.05 were displayed.**


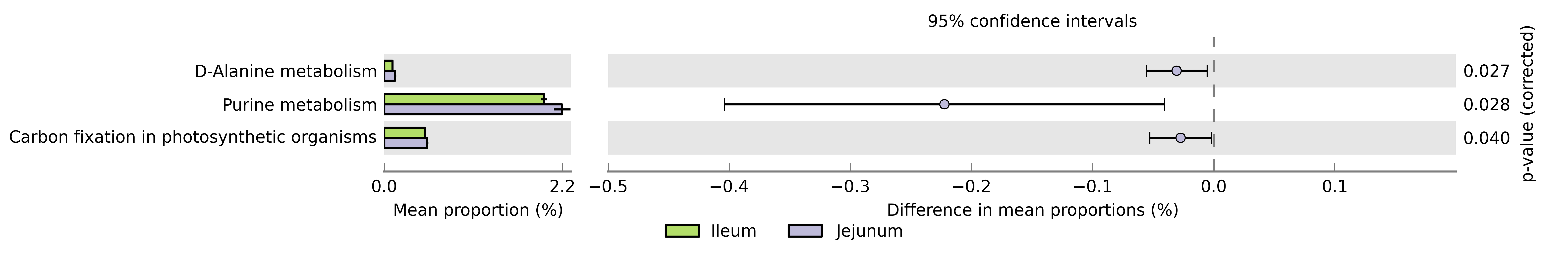

Supplement: Supplementary file 1 — Supplementary Information 1. [file 41598_2023_31684_MOESM1_ESM.docx]
