# Supplementary material for: Microbial diversity and metabolic function in duodenum, jejunum and ileum of emu (Dromaius novaehollandiae)
Source: Sci Rep. 2023 Mar 18;13:4488. doi: 10.1038/s41598-023-31684-8 (PMC10024708; doi:10.1038/s41598-023-31684-8)
Supplement: Supplementary file 2 — Supplementary Information 2. [file 41598_2023_31684_MOESM2_ESM.pdf]

Figure S7 Heatmaps representing the core bacterial diversity at (A) Class level, (B) Order level, (C) Family level, and (D) Genus level, detected from microbiome samples of the 4 emu small intestines (D1 to D4 =duodenum, J1 to J4 = jejunum, I1 to I4 = Ileum) and ceca (C1 to C4).

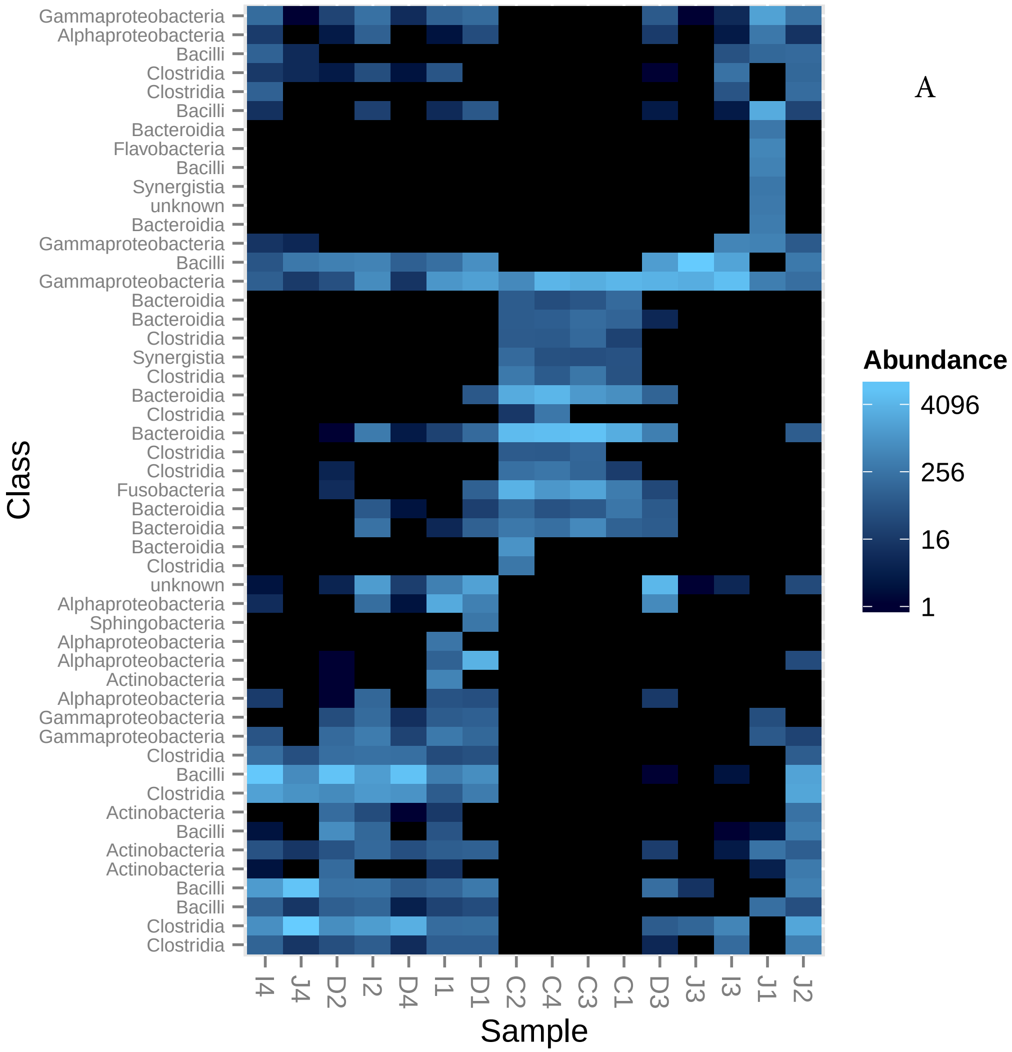

A
Abundance
4096
256
16
1
Class
Sample
Gammaproteobacteria
Alphaproteobacteria
Bacilli
Clostridia
Clostridia
Bacilli
Bacteroidia
Flavobacteria
Bacilli
Synergistia
unknown
Bacteroidia
Gammaproteobacteria
Bacilli
Gammaproteobacteria
Bacteroidia
Bacteroidia
Clostridia
Synergistia
Clostridia
Bacteroidia
Clostridia
Bacteroidia
Clostridia
Clostridia
Fusobacteria
Bacteroidia
Bacteroidia
Bacteroidia
Clostridia
unknown
Alphaproteobacteria
Sphingobacteria
Alphaproteobacteria
Alphaproteobacteria
Actinobacteria
Alphaproteobacteria
Gammaproteobacteria
Gammaproteobacteria
Clostridia
Bacilli
Clostridia
Actinobacteria
Bacilli
Actinobacteria
Actinobacteria
Bacilli
Bacilli
Clostridia
Clostridia
I4
J4
D2
I2
D4
I1
D1
C2
C4
C3
C1
D3
J3
I3
J1
J2

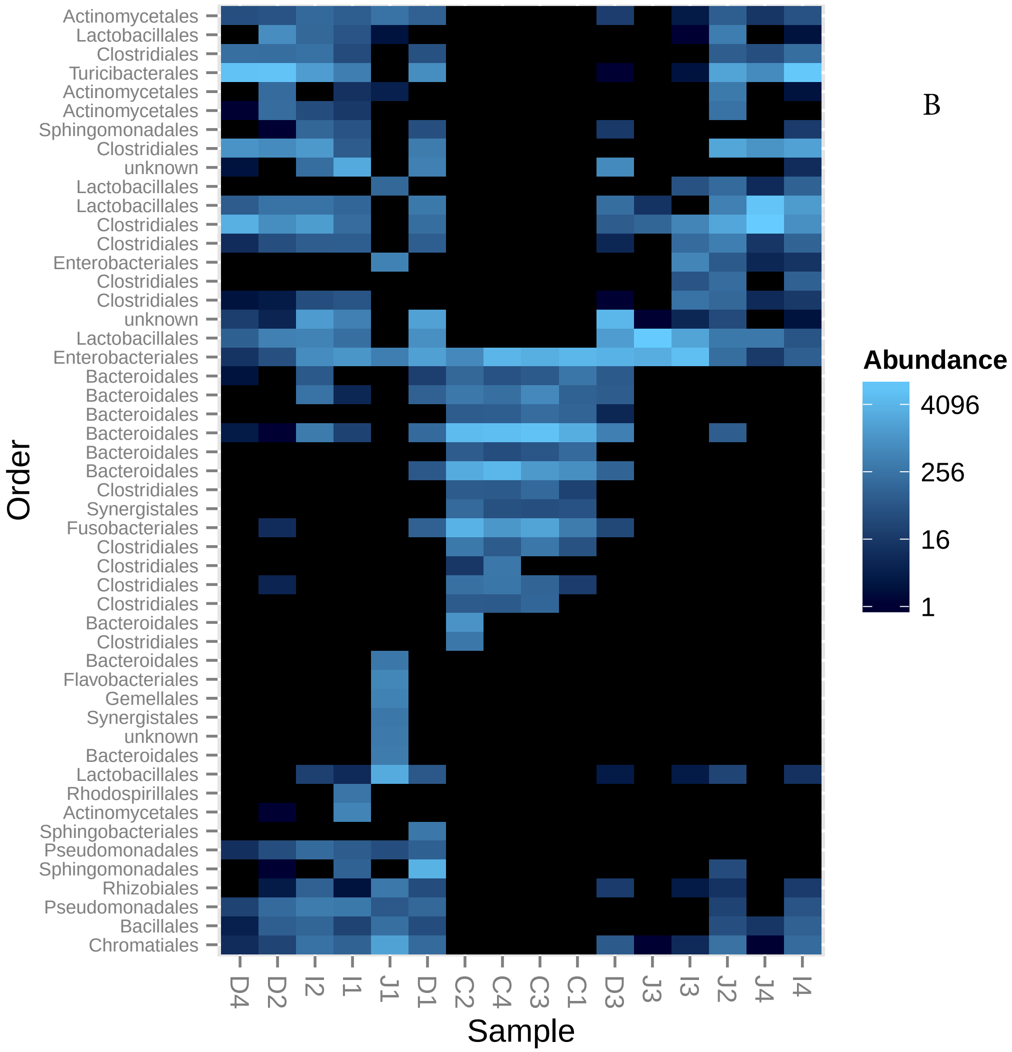
B
Abundance
4096
256
16
1
Order
Actinomycetales
Lactobacillales
Clostridiales
Turicibacterales
Actinomycetales
Actinomycetales
Sphingomonadales
Clostridiales
unknown
Lactobacillales
Lactobacillales
Clostridiales
Clostridiales
Enterobacteriales
Clostridiales
Clostridiales
unknown
Lactobacillales
Enterobacteriales
Bacteroidales
Bacteroidales
Bacteroidales
Bacteroidales
Bacteroidales
Bacteroidales
Clostridiales
Synergistales
Fusobacteriales
Clostridiales
Clostridiales
Clostridiales
Clostridiales
Bacteroidales
Clostridiales
Bacteroidales
Flavobacteriales
Gemellales
Synergistales
unknown
Bacteroidales
Lactobacillales
Rhodospirillales
Actinomycetales
Sphingobacteriales
Pseudomonadales
Sphingomonadales
Rhizobiales
Pseudomonadales
Bacillales
Chromatiales
Sample
D4
D2
I2
I1
J1
D1
C2
C4
C3
C1
D3
J3
I3
J2
J4
I4

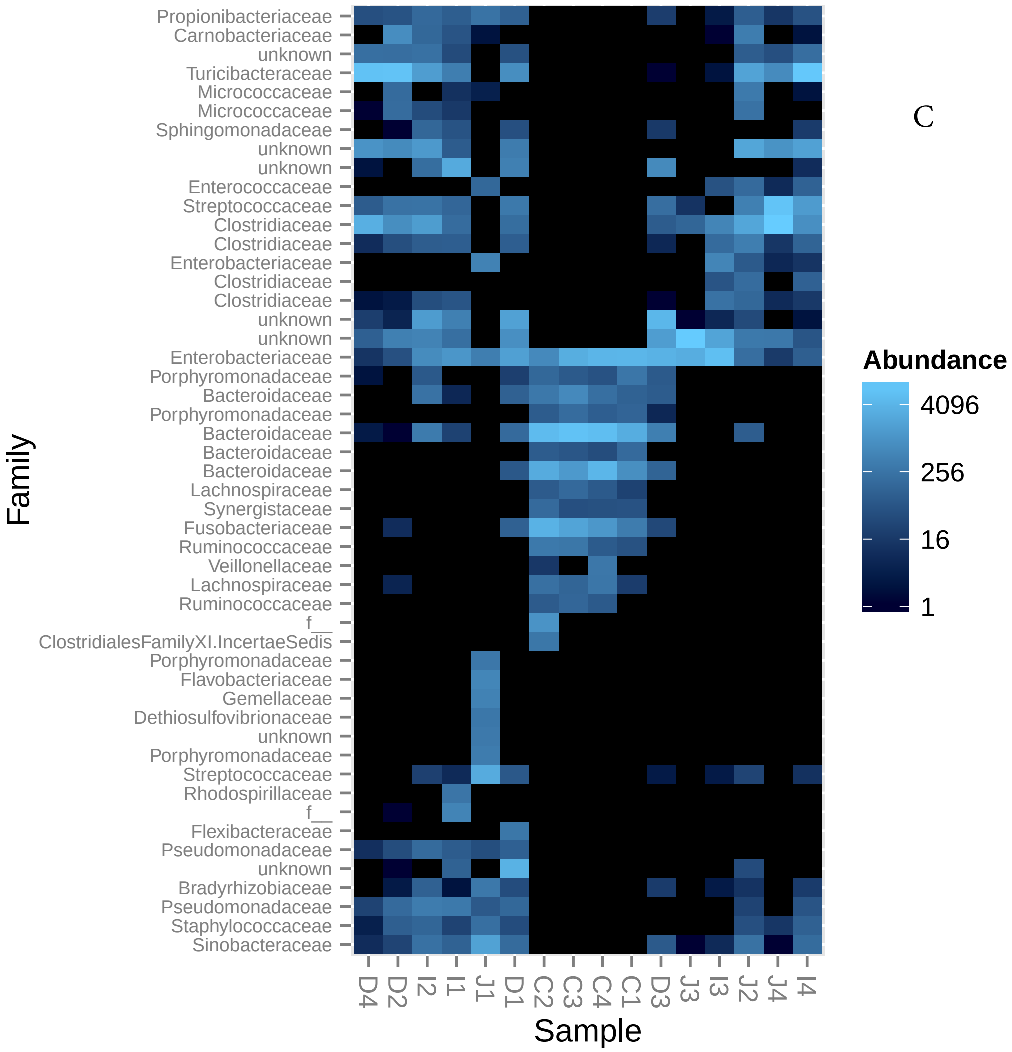
C
Family
Propionibacteriaceae
Carnobacteriaceae
unknown
Turicibacteraceae
Micrococcaceae
Micrococcaceae
Sphingomonadaceae
unknown
unknown
Enterococcaceae
Streptococcaceae
Clostridiaceae
Clostridiaceae
Enterobacteriaceae
Clostridiaceae
Clostridiaceae
unknown
unknown
Enterobacteriaceae
Porphyromonadaceae
Bacteroidaceae
Porphyromonadaceae
Bacteroidaceae
Bacteroidaceae
Bacteroidaceae
Lachnospiraceae
Synergistaceae
Fusobacteriaceae
Ruminococcaceae
Veillonellaceae
Lachnospiraceae
Ruminococcaceae
f__
ClostridialesFamilyXI.IncertaeSedis
Porphyromonadaceae
Flavobacteriaceae
Gemellaceae
Dethiosulfovibrionaceae
unknown
Porphyromonadaceae
Streptococcaceae
Rhodospirillaceae
f__
Flexibacteraceae
Pseudomonadaceae
unknown
Bradyrhizobiaceae
Pseudomonadaceae
Staphylococcaceae
Sinobacteraceae
D4
D2
I2
I1
J1
D1
C2
C3
C4
C1
D3
J3
I3
J2
J4
I4
Sample
Abundance
4096
256
16
1

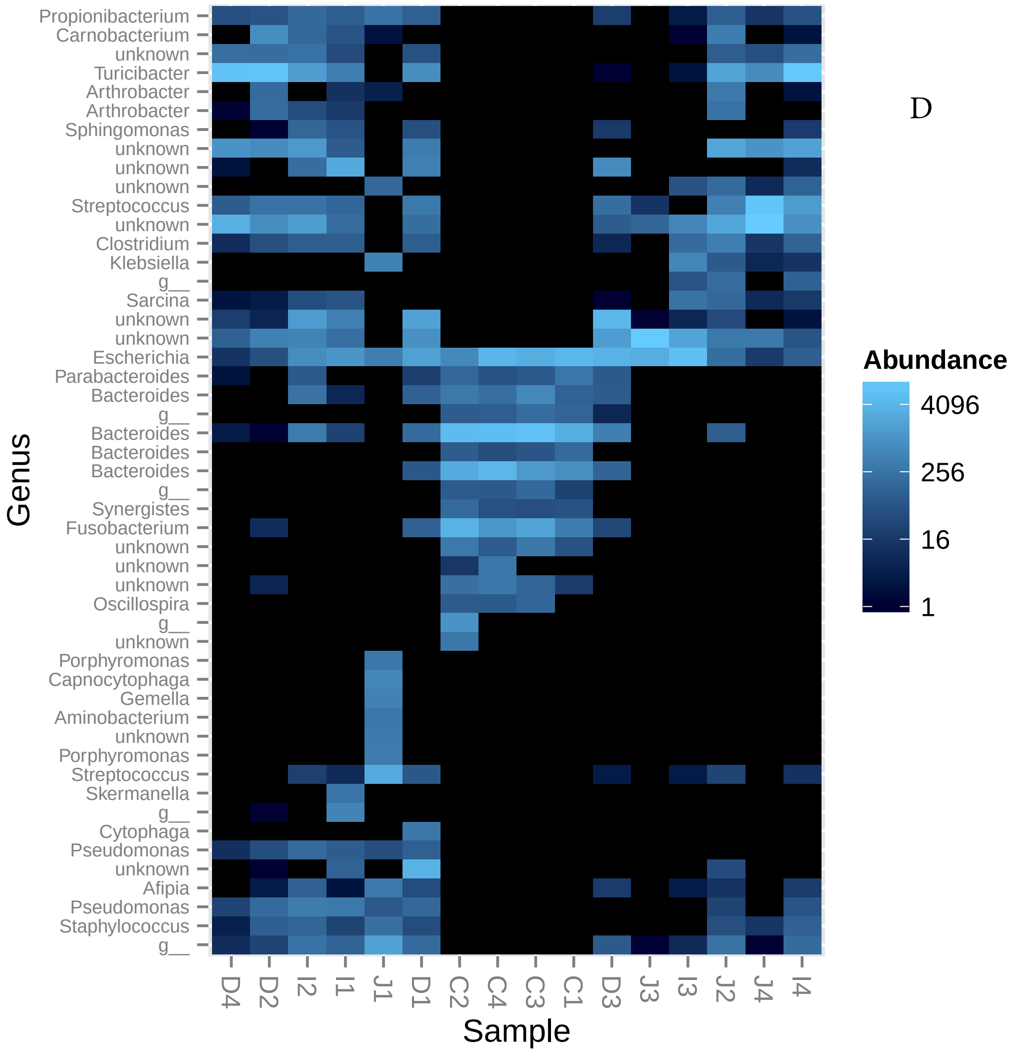

D
Genus
Sample
Abundance
4096
256
16
1
Propionibacterium
Carnobacterium
unknown
Turicibacter
Arthrobacter
Arthrobacter
Sphingomonas
unknown
unknown
unknown
Streptococcus
unknown
Clostridium
Klebsiella
g__
Sarcina
unknown
unknown
Escherichia
Parabacteroides
Bacteroides
g__
Bacteroides
Bacteroides
Bacteroides
g__
Synergistes
Fusobacterium
unknown
unknown
unknown
Oscillospira
g__
unknown
Porphyromonas
Capnocytophaga
Gemella
Aminobacterium
unknown
Porphyromonas
Streptococcus
Skermanella
g__
Cytophaga
Pseudomonas
unknown
Afipia
Pseudomonas
Staphylococcus
g__
D4
D2
I2
I1
J1
D1
C2
C4
C3
C1
D3
J3
I3
J2
J4
I4
