# Supplementary material for: Microbial diversity and metabolic function in duodenum, jejunum and ileum of emu (Dromaius novaehollandiae)
Source: Sci Rep. 2023 Mar 18;13:4488. doi: 10.1038/s41598-023-31684-8 (PMC10024708; doi:10.1038/s41598-023-31684-8)
Supplement: Supplementary file 3 — Supplementary Information 3. [file 41598_2023_31684_MOESM3_ESM.docx]

**Table S1. Relative abundance of bacterial community.**

| **Taxon** | **Duodenum** | | | | **Jejunum** | | | | **Ileum** | | | | **Cecum** | | | |
| --- | --- | --- | --- | --- | --- | --- | --- | --- | --- | --- | --- | --- | --- | --- | --- | --- |
|  | **D1** | **D2** | **D3** | **D4** | **J1** | **J2** | **J3** | **J4** | **I1** | **I2** | **I3** | **I4** | **C1** | **C2** | **C3** | **C4** |
| Unclassified Bacteria | 0.01% | 0.03% | 0.03% | 0.02% | 0.00% | 0.17% | 0.01% | 0.01% | 1.15% | 0.51% | 0.35% | 0.00% | 1.10% | 1.11% | 1.04% | 0.90% |
| Acidobacteria | 0.19% | 0.00% | 0.00% | 0.00% | 0.00% | 0.00% | 0.00% | 0.00% | 2.06% | 0.00% | 0.00% | 0.00% | 0.00% | 0.00% | 0.00% | 0.00% |
| Actinobacteria | **2.51%** | **4.63%** | 0.28% | 0.42% | **5.64%** | **7.61%** | 0.00% | 0.09% | **17.76%** | **3.71%** | 0.09% | 0.72% | 0.05% | 0.06% | 0.04% | 0.10% |
| BRC1 | 0.00% | 0.00% | 0.00% | 0.00% | 0.00% | 0.00% | 0.00% | 0.00% | 0.05% | 0.00% | 0.00% | 0.00% | 0.00% | 0.00% | 0.00% | 0.00% |
| Bacteroidetes | **6.73%** | **0.44%** | **6.98%** | **0.05%** | **20.76%** | **1.46%** | 0.00% | 0.01% | 2.59% | 6.38% | 0.00% | 0.71% | **47.35%** | **59.85%** | **57.78%** | **60.66%** |
| Chloroflexi | 0.00% | 0.26% | 0.02% | 0.01% | 0.00% | 0.43% | 0.00% | 0.00% | 1.85% | 0.00% | 0.00% | 0.00% | 0.00% | 0.00% | 0.00% | 0.00% |
| Cyanobacteria | 0.02% | 0.03% | 0.00% | 0.00% | 0.00% | 0.00% | 0.00% | 0.02% | 0.53% | 0.09% | 0.16% | 0.00% | 0.00% | 0.00% | 0.00% | 0.00% |
| Elusimicrobia | 0.00% | 0.03% | 0.00% | 0.00% | 0.00% | 0.00% | 0.00% | 0.00% | 0.00% | 0.00% | 0.00% | 0.00% | 0.00% | 0.00% | 0.00% | 0.00% |
| Firmicutes | **21.90%** | **91.07%** | **16.24%** | **98.74%** | **35.63%** | **81.88%** | **77.74%** | **99.68%** | **13.80%** | **54.93%** | **33.69%** | **94.32%** | **2.88%** | **11.00%** | **7.30%** | **6.68%** |
| Fusobacteria | 0.76% | 0.06% | 0.26% | 0.00% | 0.00% | 0.00% | 0.00% | 0.00% | 0.00% | 0.00% | 0.00% | 0.00% | 3.84% | **21.66%** | **12.75%** | **6.75%** |
| Gemmatimonadetes | 0.00% | 0.00% | 0.00% | 0.00% | 0.00% | 0.00% | 0.00% | 0.00% | 0.02% | 0.00% | 0.00% | 0.00% | 0.00% | 0.00% | 0.00% | 0.00% |
| Lentisphaerae | 0.00% | 0.00% | 0.00% | 0.00% | 0.00% | 0.00% | 0.00% | 0.00% | 0.00% | 0.00% | 0.00% | 0.00% | 0.00% | 0.05% | 0.01% | 0.08% |
| Planctomycetes | 0.00% | 0.01% | 0.00% | 0.00% | 0.00% | 0.00% | 0.00% | 0.00% | 0.00% | 0.00% | 0.00% | 0.00% | 0.00% | 0.00% | 0.00% | 0.00% |
| Proteobacteria | **67.30%** | **3.44%** | **76.07%** | **0.77%** | **33.74%** | **8.45%** | **22.26%** | 0.18% | **59.77%** | **34.38%** | **65.71%** | **4.25%** | **44.19%** | **5.18%** | **20.73%** | **24.46%** |
| SC4 | 0.04% | 0.00% | 0.00% | 0.00% | 0.00% | 0.00% | 0.00% | 0.00% | 0.02% | 0.00% | 0.00% | 0.00% | 0.00% | 0.00% | 0.00% | 0.00% |
| Spirochaetes | 0.00% | 0.00% | 0.00% | 0.00% | 1.66% | 0.00% | 0.00% | 0.00% | 0.06% | 0.00% | 0.00% | 0.00% | 0.00% | 0.00% | 0.00% | 0.00% |
| Synergistetes | 0.00% | 0.00% | 0.01% | 0.00% | 2.50% | 0.00% | 0.00% | 0.00% | 0.02% | 0.00% | 0.00% | 0.00% | 0.58% | 1.07% | 0.30% | 0.34% |
| Tenericutes | 0.00% | 0.00% | 0.10% | 0.00% | 0.07% | 0.00% | 0.00% | 0.00% | 0.15% | 0.00% | 0.00% | 0.00% | 0.01% | 0.01% | 0.03% | 0.01% |
| Thermi | 0.55% | 0.01% | 0.01% | 0.00% | 0.00% | 0.00% | 0.00% | 0.00% | 0.19% | 0.00% | 0.00% | 0.00% | 0.00% | 0.00% | 0.00% | 0.00% |
| Verrucomicrobia | 0.00% | 0.00% | 0.00% | 0.00% | 0.00% | 0.00% | 0.00% | 0.00% | 0.00% | 0.00% | 0.00% | 0.00% | 0.00% | 0.01% | 0.02% | 0.01% |
| Total | 100% | 99.9% | 100% | 100% | 100% | 100% | 100% | 100% | 100% | 100% | 100% | 100% | 100% | 100% | 100% | 100% |

**Table S2.** Ranking of significant difference in metabolic function among the 4 intestinal segments

| Metabolic function | Duodenum | Jejunum | Ileum | Cecum |
| --- | --- | --- | --- | --- |
| Adipocytokine signalling pathway |  | 2 |  | 1 |
| Alanine, aspartate and glutamate metabolism | 2 | 2 | 2 | 1 |
| Amino sugar & nucleotide sugar metabolism | 2 |  | 2 | 1 |
| Antigen processing and presentation |  | 2 | 2 | 1 |
| Benzoate degradation |  | 1 |  | 2 |
| Biotin metabolism |  | 2 | 2 | 1 |
| Biosynthesis of vancomycin group antibiotics | 3 | 2 | 2 | 1 |
| Butanoate metabolism |  | 1 |  | 2 |
| Butirosin and neomycin biosynthesis | 2 |  | 2 | 1 |
| Carbon fixation and photosynthetic organisms | 2 |  |  | 1 |
| Carbohydrate digestion and absorption |  | 2 | 2 | 1 |
| Carbohydrate metabolism |  | 2 |  | 1 |
| Cell division |  |  | 2 | 1 |
| Chloroalkane and chloroalkene degradation |  | 1 | 1 | 2 |
| Drug metabolism – cytochrome P450 |  |  | 1 | 2 |
| D-alanine metabolism | 2 | 1 | 1 | 3 |
| Dioxin degradation |  | 1 |  | 2 |
| Energy metabolism |  | 2 | 2 | 1 |
| Fatty acid biosynthesis |  | 1 |  | 2 |
| Galactose metabolism | 2 |  | 2 | 1 |
| General function prediction only | 2 |  | 2 | 1 |
| Glutathione metabolism |  |  | 1 | 2 |
| Glutametergic synapse | 2 |  |  | 1 |
| Glycosphingolipid biosynthesis – ganglio series | 2 | 2 | 2 | 1 |
| Glycosaminoglycan degradation | 2 | 2 | 2 | 1 |
| Glycosphingolipid biosynthesis – globo series | 2 | 2 | 2 | 1 |
| Insulin signalling pathway |  | 2 | 2 | 1 |
| Isoquinolin alkaloid biosynthesis | 2 |  | 2 | 1 |
| Lipid metabolism |  | 1 |  | 2 |
| Lipoic acid metabolism |  |  | 2 | 1 |
| Lipopolysaccharide biosynthesis protein |  | 2 |  | 1 |
| Membrane & intracellular structural molecules |  |  | 2 | 1 |
| Metabolism of co-factors and vitamins | 1 |  | 1 | 2 |
| Methane metabolism | 2 |  |  | 1 |
| NOD-like receptor signalling pathway |  | 2 |  | 1 |
| Nitrogen metabolism |  | 2 |  | 1 |
| Other glycan degradation | 2 | 2 | 2 | 1 |
| Other ion-coupled transporters |  | 2 | 2 | 1 |
| Phenopropanoid biosynthesis |  | 2 |  | 1 |
| Phosphonate and phosphinate metabolism | 3 | 2 | 2 | 1 |
| Protein digestion and absorption | 2 | 2 | 2 | 1 |
| Polyketide sugar unit biosynthesis | 2 | 2 | 2 | 1 |
| Purine metabolism | 3 | 2 | 2 | 1 |
| Restriction enzyme | 2 | 2 | 2 | 1 |
| Retinol (Vitamin A1) metabolism |  |  | 1 | 2 |
| Riboflavin metabolism | 1 |  |  | 2 |
| Secondary bile acid biosynthesis |  |  | 2 | 1 |
| Sphingolipid metabolism | 2 | 2 | 2 | 1 |
| Steroid hormone biosynthesis | 2 | 2 | 2 | 1 |
| Streptomycine biosynthesis | 2 | 2 | 2 | 1 |
| Styrene degradation |  | 1 |  | 2 |
| Sulfur metabolism |  |  | 2 | 1 |
| Translation protein |  | 1 |  | 2 |
| Tyrosine metabolism |  | 1 |  | 2 |
| Vitamin B6 metabolism |  | 2 | 2 | 1 |
| Xylene degradation |  | 1 | 1 | 2 |
| Zeatin biosynthesis | 2 | 2 | 2 | 1 |

Across each line, significant difference in microbiota metabolic functions among the intestinal segments:

1 > 2 > 3; Blanks = no significant difference between that small intestine segment and the other intestinal segments.
